# Lugdunin amplifies innate immune responses in the skin in synergy with host- and microbiota-derived factors

**DOI:** 10.1038/s41467-019-10646-7

**Published:** 2019-06-21

**Authors:** Katharina Bitschar, Birgit Sauer, Jule Focken, Hanna Dehmer, Sonja Moos, Martin Konnerth, Nadine A. Schilling, Stephanie Grond, Hubert Kalbacher, Florian C. Kurschus, Friedrich Götz, Bernhard Krismer, Andreas Peschel, Birgit Schittek

**Affiliations:** 1 Department of Dermatology, University of Tübingen, Liebermeisterstraße 25, 72076 Tübingen, Germany; 2 Institute for Molecular Medicine, University Medical Center of the Johannes Gutenberg-University Mainz, Langenbeckstraße 1, 55131 Mainz, Germany; 3 Institute of Organic Chemistry, University of Tübingen, Auf der Morgenstelle 18, 72076 Tübingen, Germany; 4 Interfaculty Institute of Biochemistry, University of Tübingen, Ob dem Himmelreich 7, 72074 Tübingen, Germany; 5 Department of Dermatology, Heidelberg University Hospital, Im Neuenheimer Feld 440, 69120 Heidelberg, Germany; 6 Interfaculty Institute of Microbiology and Infection Medicine, Microbial Genetics, University of Tübingen, Auf der Morgenstelle 28, 72076 Tübingen, Germany; 7 Interfaculty Institute of Microbiology and Infection Medicine, Infection Biology, University of Tübingen, Auf der Morgenstelle 28, 72076 Tübingen, Germany; 8 German Centre for Infection Research (DZIF), Partner Site Tübingen, Auf der Morgenstelle 28, 72076 Tübingen, Germany

**Keywords:** Immunology, Microbiology, Diseases

## Abstract

Recently our groups discovered lugdunin, a new cyclic peptide antibiotic that inhibits S*taphylococcus aureus* epithelial colonization in humans and rodents. In this work, we analyzed its immuno-modulatory and antimicrobial potential as a single agent or in combination with other microbiota- or host-derived factors. We show that pretreatment of primary human keratinocytes or mouse skin with lugdunin in combination with microbiota-derived factors results in a significant reduction of *S. aureus* colonization. Moreover, lugdunin increases expression and release of LL-37 and CXCL8/MIP-2 in human keratinocytes and mouse skin, and results in the recruitment of monocytes and neutrophils in vivo, both by a TLR/MyD88-dependent mechanism. Interestingly, *S. aureus* elimination by lugdunin is additionally achieved by synergistic antimicrobial activity with LL-37 and dermcidin-derived peptides. In summary, our results indicate that lugdunin provides multi-level protection against *S. aureus* and may thus become a promising treatment option for *S. aureus* skin infections in the future.

## Abstract (web summary)

Lugdunin is a peptide antibiotic produced by the skin commensal *Staphylococcus lugdunensis*. Here, the authors show that lugdunin reduces *Staphylococcus aureus* colonization in human keratinocytes and mouse skin by inducing the expression of human LL-37 and recruitment of monocytes and neutrophils.

## Introduction

Skin is a challenging habitat for bacteria with conditions, including dryness, low nutrient availability, high salt concentrations, and low pH, as well as the presence of host antimicrobial peptides (AMPs) and lipids^1^. Nevertheless, human skin is populated by a complex microbiota whose composition is mainly determined by the ecologic feature of the body site^2,3^, but is also largely influenced by host- and bacteria-derived factors. During steady state, a constant interplay among them allows for colonization with commensal microorganisms, while at the same time pathogenic microorganisms such as *Staphylococcus aureus* can be efficiently prevented from persisting.

Characteristic changes in the composition of skin microbial consortia have been associated with chronic skin disorders such as atopic dermatitis (AD)^4^. Usually, *S. aureus* can hardly be found on healthy skin and only in 30% of the human population in the anterior nares^5^, but it is abundant on inflamed and non-inflamed skin of AD patients^4^. Interestingly, overabundance of cutaneous *S. aureus*, especially during AD flares, is associated with loss of microbiome diversity, indicating that the skin microbiome shapes *S. aureus* skin colonization^3,4^. However, the mechanisms that are used by the skin microbiota during steady state to prevent colonization by *S. aureus* still remain elusive. Only recently we showed that the skin commensal *S. epidermidis* is able to amplify the innate immune response of the skin against pathogens by creating a protective environment, which ultimately leads to reduction of *S. aureus* colonization^6^.

Apart from occupying space and triggering innate immune responses, the microbiota shields our skin from pathogen colonization by the release of specific AMPs called bacteriocins that can directly act on competing bacteria. Commensal-produced factors were shown to directly inhibit *S. aureus* growth^7,8^. Coagulase-negative staphylococci are frequent producers of post-translationally modified lanthionine-containing bacteriocins (lantibiotics)^9^. Recently, we discovered a novel peptide antibiotic produced by the nasal and skin commensal *Staphylococcus lugdunensis*, named lugdunin^10^. This newly discovered compound is a thiazolidine-containing cyclic peptide antibiotic, which is non-ribosomally synthesized and belongs to a new class of antibacterials^10^. Lugdunin displays potent antimicrobial activity against a wide range of Gram-positive bacteria including *S. aureus*. Importantly, humans who carry *S. lugdunensis* have a 6-fold lower risk of *S. aureus* nasal carriage^10^.

We previously showed that lugdunin efficiently reduces *S. aureus* skin and nasal colonization^10^; however, the inhibitory mechanism is not completely understood. In addition to direct killing, lugdunin might reduce *S. aureus* colonization indirectly by modulation of skin defense mechanisms or by a combination of both. In fact, potential immuno-modulatory properties of bacterial AMPs such as lugdunin have rarely been investigated. In contrast, it is well established that host-derived AMPs such as the human β-defensins (HBD) 1–3 and the cathelicidin LL-37 are not only able to kill a diverse set of microorganisms but also modulate innate immune responses^11^. Here, we demonstrate that lugdunin prevents *S. aureus* colonization not only by a direct killing mechanism but also by additionally triggering increased innate defense of epithelial cells. Furthermore, synergistic and/or antagonistic activities between bacterial- and host-derived AMPs further contribute to *S. aureus* colonization resistance, which might be a common phenomenon in the complex interplay of microbes and host.

## Results

### Lugdunin amplifies commensal-induced *S. aureus* protection

**Fig. 1:**
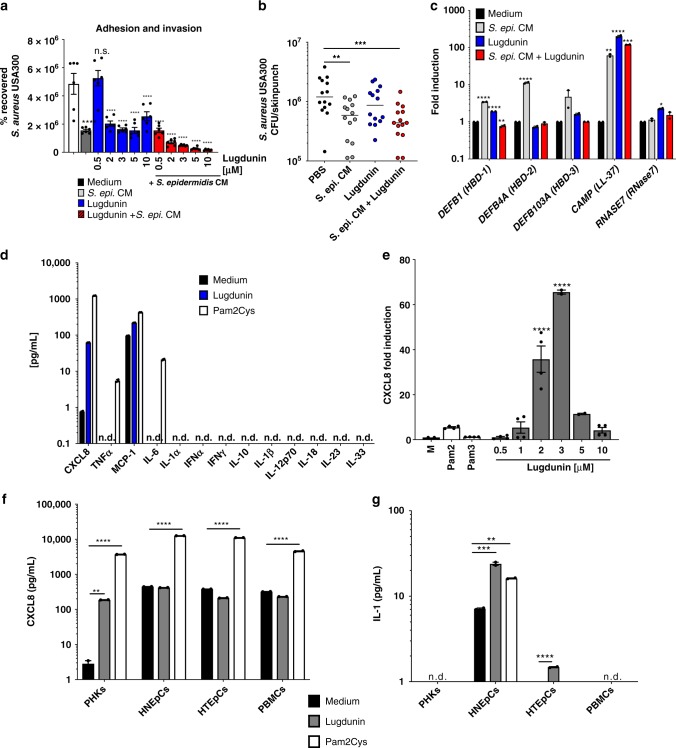
Lugdunin sensitizes epithelial cells for innate immune defense. **a** Primary human keratinocytes (PHKs) were pretreated with *S. epidermidis* conditioned medium (CM), indicated lugdunin concentrations, or the combination of both for 20 h. Subsequently, cells were infected with *S. aureus* for 1.5 h followed by cell lysis and determination of colony-forming units (CFUs). Shown is one representative experiment of three independent experiments with six technical replicates  ± s.e.m. n.s., not significant. **b** Dorsal skin of mice was pretreated with *S. epidermidis* CM, 1.5 µg lugdunin alone or in combination with *S. epidermidis* CM for 24 h. Subsequently, *S. aureus* CFUs were determined. Horizontal lines represent the mean of each group ± s.e.m. **c** PHKs were treated with 2 μM lugdunin or with *S. epidermidis* CM alone or in combination for 20 h and subsequently expression of indicated antimicrobial peptides (AMPs) (respective protein names in brackets) was analyzed and normalized to actin. Shown is one representative experiment of three independent experiments with two technical replicates ± s.e.m. **d** PHKs were treated with 2 µM lugdunin or 100 ng/mL Pam2Cys for 5 h and subsequently the concentration of indicated cytokines in the supernatant was analyzed by LEGENDplex^TM^ (BioLegend). Shown is one representative experiment of three independent experiments with two technical replicates ± s.e.m. **e** PHKs were treated with increasing concentrations of lugdunin or 100 ng/mL Pam2Cys or Pam3Cys for 5 h and subsequently expression of chemokine (C-X-C motif) ligand 8 (CXCL8) was analyzed and normalized to actin. Shown is one representative experiment of three independent experiments, each with two technical replicates ± s.e.m. **f**, **g** Indicated cells were treated with 2 µM lugdunin or 100 ng/mL Pam2Cys as a positive control for 5 h and subsequently the concentration of CXCL8 (**f**) and interleukin-1α (IL-1α) (**g**) in the supernatant was analyzed by LEGENDplex^TM^ (BioLegend) and enzyme-linked immunosorbent assay (ELISA) (R&D Systems). Shown is one representative experiment of three independent experiments with two technical replicates ± s.e.m. Significant differences to control treatments were analyzed by ordinary one-way analysis of variance (ANOVA) followed by Dunnett’s multiple comparisons test (*P < 0.05; **P < 0.01; ***P < 0.001; ****P < 0.0001). n.d. = not detected. Source data are provided as a Source Data file

Recently, we showed that the novel peptide antibiotic lugdunin, produced by the nasal commensal *S. lugdunensis*, effectively interferes with *S. aureus* epithelial colonization in rodents and humans^10^. On the one hand, this results from the direct bactericidal effect of lugdunin against *S. aureus*, but on the other hand, there might be an additional mechanism mediated by immune conditioning of epithelial cells by lugdunin. We proposed that lugdunin might sensitize epithelial cells towards an enhanced innate response, which prevents *S. aureus* colonization similar to the protective effect mediated by secreted factors of the skin commensal *S. epidermidis*, which was recently described by our group^6^. Therefore, we first tested whether pretreatment of primary human keratinocytes (PHKs) as well as mouse skin with lugdunin interferes with *S. aureus* colonization. As it is shown in Fig. 1a pretreatment of PHKs with lugdunin alone at concentrations of 2 µM and above significantly reduced the number of adhering *S. aureus*. Similarly, treatment of mouse skin with lugdunin alone resulted in a slight, but not significant, reduction of colonizing *S. aureus* (Fig. 1b). These reductions in *S. aureus* colonization were significantly enhanced when lugdunin was combined with *S. epidermidis* conditioned medium (CM) (Fig. 1a, b). These data indicate that lugdunin is able to sensitize keratinocytes towards a protective response against *S. aureus* skin colonization and that lugdunin and factors produced by other skin commensals act in concert.

### Lugdunin induces LL-37 and CXCL8 in keratinocytes

To elucidate the mechanism of the lugdunin-induced protective response, we analyzed whether lugdunin is able to induce the expression of AMPs or pro-inflammatory cytokines in PHKs, either alone or in combination with *S. epidermidis* CM. PHKs express a basal level of the β-defensins HBD-1, HBD-2, and HBD-3, as well as LL-37 and RNase7^12^. *Staphylococcus epidermidis* CM was able to significantly induce the expression of HBD-1, HBD-2, and LL-37 (Fig. 1c), confirming our previous studies^13^. More importantly, lugdunin treatment of PHKs alone significantly induced expression and release of LL-37 in a dose-dependent manner as well as expression of HBD-1 and RNase7 (Fig. 1c and Supplementary Fig. 1). Surprisingly, combined treatment of PHKs with *S. epidermidis* CM and lugdunin abolished the effect of *S. epidermidis* CM on the induction of the respective AMPs, except for LL-37 (Fig. 1c). Next, we analyzed, whether lugdunin is able to induce the secretion of a set of 13 different pro-inflammatory cytokines and chemokines in PHKs. Interestingly, increasing lugdunin concentrations up to 3 µM specifically induced expression and release of chemokine (C-X-C motif) ligand 8 (CXCL8) in PHKs (Fig. 1d, e and Supplementary Fig. 1), while higher concentrations did not induce CXCL8 production (Fig. 1e and Supplementary Fig. 1). Conversely, expression and release of LL-37 increased with higher lugdunin concentrations (Supplementary Fig. 1). Furthermore, we topically applied lugdunin on a human 3D skin equivalent and confirmed the lugdunin-induced LL-37 and CXCL8 secretion (Supplementary Fig. 1). Additionally, expression of LL-37 in keratinocytes was confirmed by immunohistochemical stainings of mouse skin sections upon epicutaneous application of lugdunin and *S. lugdunensis* (Supplementary Fig. 1). Of note, synthetic lugdunin resulted in similar CXCL8 expression levels in PHKs as the natural lugdunin. However, the non-antimicrobial *N*-acetyl-lugdunin, on the other hand, did not induce CXCL8 expression in PHKs (Supplementary Fig. 1). Notably, lugdunin was also able to induce CXCL8 expression in primary human nasal and tracheal epithelial cells (HNEpCs and HTEpCs), as well as in peripheral blood mononuclear cells (PBMCs) (Supplementary Fig. 1), but with a much lower efficacy. Of note, basal protein levels of CXCL8 in these cells were already higher compared to PHKs and could not be further increased by lugdunin treatment (Fig. 1f). In HNEpCs and HTEpCs, but not in PHKs and PBMCs, lugdunin was also able to induce the release of interleukin-1α (IL-1α), another important pro-inflammatory cytokine (Fig. 1d, g). Therefore, lugdunin induces different sets of pro-inflammatory cytokines in a cell-type-specific manner. Noteworthy, lugdunin treatment was not toxic to the cell types analyzed (Supplementary Fig. 1). In summary, our data indicate that lugdunin is able to induce the expression of LL-37 and pro-inflammatory cytokines in PHKs, which might modulate the response of PHKs towards *S. aureus* skin colonization.

### Lugdunin-induced cytokine production is TLR/MyD88 dependent

**Fig. 2:**
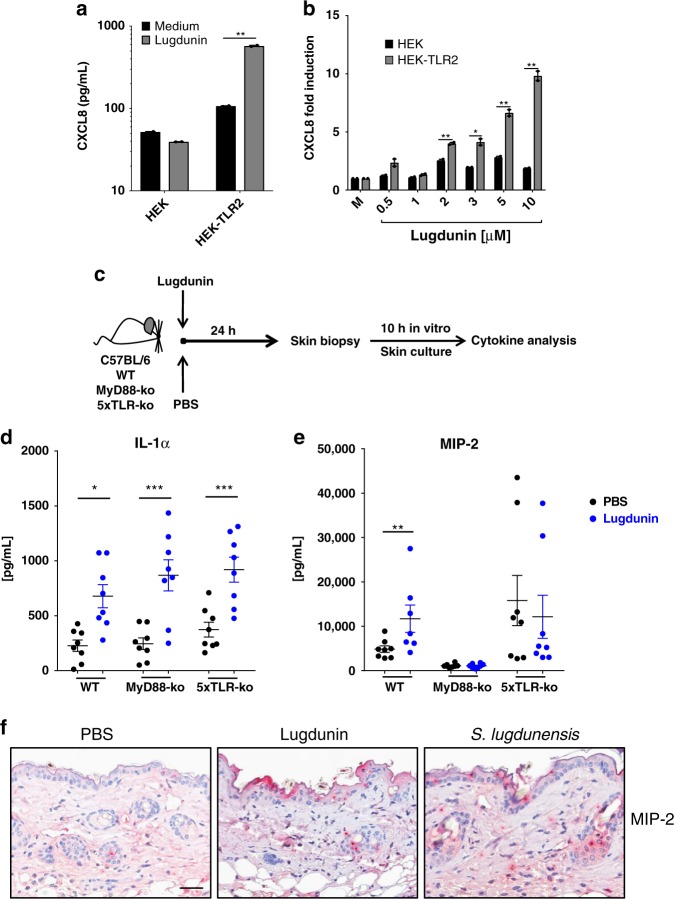
Induction of chemokine (C-X-C motif) ligand 8/macrophage inflammatory protein-2 (CXCL8/MIP-2) by lugdunin is TLR2/MyD88-dependent. **a** HEK-control or HEK-TLR2 cells were treated with 2 μM lugdunin for 5 h and subsequently the CXCL8 concentration in the supernatant was analyzed by LEGENDplex^TM^ (BioLegend). Shown is one representative experiment of three independent experiments with two technical replicates ± s.e.m. **b** HEK-control or HEK-TLR2 cells were treated with increasing concentrations of lugdunin for 5 h and subsequently expression of CXCL8 was analyzed and normalized to actin. Shown is one representative experiment of three independent experiments with two technical replicates ± s.e.m. **c** Schematic overview of the mouse experiments: 6–8-week-old female C5BL/6 WT, MyD88-knockout (ko), or 5xTLR-ko mice were epicutaneously treated with 1.5 µg lugdunin or phosphate-buffered saline (PBS) as a control. After 24 h, mice were euthanized, 4 mm skin punches were taken, and further cultured in vitro for 10 h followed by cytokine analysis of the culture supernatant by LEGENDplex^TM^ (BioLegend). **d**, **e** Shown are the mean concentrations of IL-1α (**d**) or MIP-2 (**e**) in the skin culture supernatant of two skin punches from four mice each ± s.e.m. Significant differences to control treatments were analyzed by an unpaired two-tailed *t* test (*P < 0.05; **P < 0.01; ***P < 0.001). **f** Representative MIP-2-stained paraffin-embedded mouse skin sections. Scale bar, 100 µM. Source data are provided as a Source Data file

CXCL8 expression can be induced in PHKs and other cell types by activation of the Toll-like receptor 2 (TLR2) signaling pathway^14^. Indeed, as shown in Fig. 1d, stimulation with the TLR2 ligand Pam2Cys led to a strong induction of CXCL8 secretion in PHKs, roughly 10-fold higher than lugdunin-induced CXCL8 secretion. Lugdunin is a thiazolidine-containing cyclic peptide and as such has not been described to activate TLR2 signaling. Therefore, we analyzed the potential role of TLR2 in lugdunin-induced CXCL8 release. Since PHKs constitutively express TLR2^15^, we used HEK293 cells, which do not express TLR2, and HEK293-TLR2 cells, which were transfected with a TLR2-containing plasmid leading to surface expression of TLR2^16^. We treated both cell types with lugdunin as well as with the TLR2 ligands Pam2Cys and Pam3Cys as positive controls and analyzed expression along with protein levels of CXCL8. Pam2Cys and Pam3Cys treatment of HEK cells induced CXCL8 expression and release in a TLR2-dependent way (Supplementary Fig. 2). Interestingly, lugdunin-induced CXCL8 expression and protein release in HEK cells was also TLR2 dependent (Fig. 2a, b) in a concentration-dependent fashion (Fig. 2b). Induction levels were, however, 1000-fold lower compared to Pam2Cys/Pam3Cys-mediated TLR2 activation (Fig. 2a and Supplementary Fig. 2).

To further investigate the involvement of TLRs, we analyzed whether lugdunin is able to induce a set of cytokines in mouse skin similar to PHKs. Therefore, we epicutaneously treated C57BL/6 mouse skin with lugdunin or phosphate-buffered saline (PBS) as a control for 24 h and determined the levels of pro-inflammatory cytokines in the skin (Fig. 2c–e and Supplementary Fig. 2). Interestingly, we found that specifically IL-1α and macrophage inflammatory protein-2 (MIP-2), the functional mouse homolog of human CXCL8^17^, were induced in mouse skin by lugdunin treatment (Fig. 2d, e). Levels of other cytokines such as monocyte chemotactic protein-1, granulocyte–macrophage colony-stimulating factor, tumor necrosis factor-α, IL-6, and interferon-γ did not show significant differences compared to the control group, except for the anti-inflammatory cytokine IL-10 (Supplementary Fig. 2). To further confirm the production of MIP-2 in mouse skin, we performed immunohistochemical analyses of mouse skin sections upon epicutaneous treatment with lugdunin or *S. lugdunensis*. Similarly to LL-37 (Supplementary Fig. 1), we could confirm MIP-2 production by keratinocytes in the epidermis and the hair follicles, which also correlates with the main locations for *S. lugdunensis* colonization in vivo (Fig. 2f and Supplementary Fig. 2). To analyze the importance of TLR and MyD88 signaling in lugdunin-mediated cytokine induction, we additionally analyzed cytokine levels upon lugdunin treatment in the skin of mice deficient for MyD88 (MyD88-knockout (ko)) or for TLR2, TLR3, TLR4, TLR7, and TLR9 (5xTLR-ko). Surprisingly, expression of most cytokines was not significantly different upon lugdunin treatment compared to wild-type (WT) control mice (Supplementary Fig. 2). Lugdunin was still able to induce IL-1α in mouse skin lacking MyD88 or TLRs (5xTLR-ko) (Fig. 2d). However, lugdunin-dependent induction of MIP-2 was completely impaired in mice lacking MyD88 and reduced in 5xTLR-ko mice (Fig. 2e). In summary, these data indicate that lugdunin induces CXCL8/MIP-2 in keratinocytes by a TLR/MyD88-dependent mechanism.

### Epicutaneous lugdunin recruits phagocytic cells

**Fig. 3:**
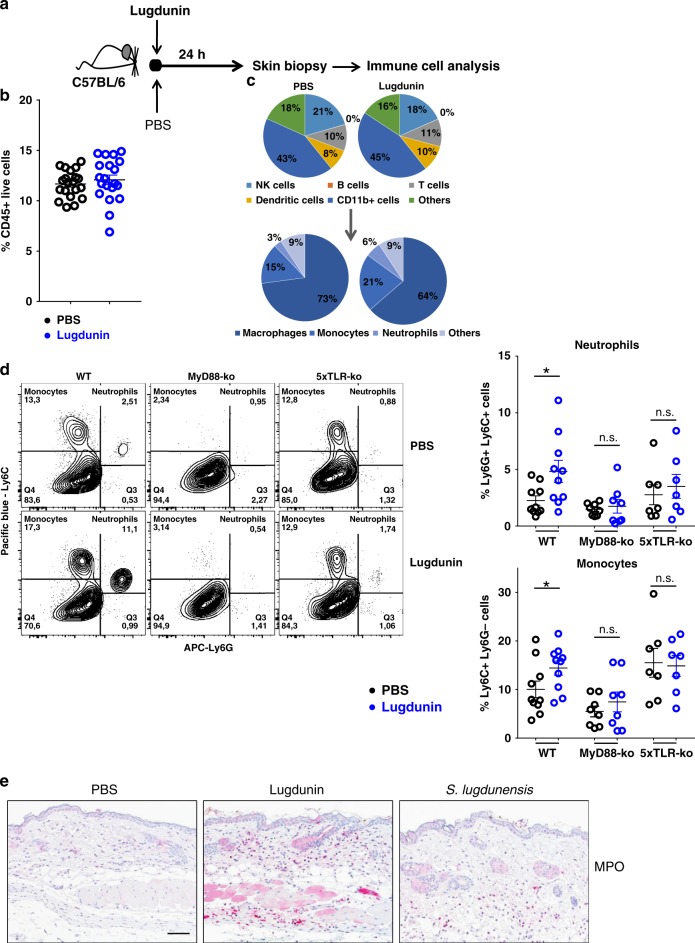
Epicutaneous lugdunin recruits phagocytic cells. **a** Schematic overview of the mouse experiments: 6–8-week-old female C5BL/6 wild-type (WT), MyD88-ko, or 5xTLR-ko mice were epicutaneously treated with 1.5 µg lugdunin or phosphate-buffered saline (PBS) as a control. After 24 h, mice were euthanized, immune cells were isolated from treated skin areas, and immune cell composition was analyzed by flow cytometry. **b** Shown is the mean percentage of CD45+  live cells in mouse skin of 10 C57BL/6 WT mice ± s.e.m. One mouse is represented as two dots analyzed by two different stainings. **c** Pie charts show the mean percentage of the different immune cell subsets in the skin of 10 WT mice after 24 h of PBS or lugdunin treatment. **d** Shown are representative flow cytometry data (left panel) and the mean percentage of neutrophils (Ly6C+Ly6G+) and monocytes (Ly6C+Ly6G−) pregated on CD11b+CD45+ live cells (see Supplementary Fig. 3a, f for the gating strategy) in mouse skin ± s.e.m. One dot represents one mouse. *P < 0.05. **e** Representative myeloperoxidase (MPO)-stained paraffin-embedded mouse skin sections. Scale bar, 100 µM. Source data are provided as a Source Data file

Induction of CXCL8/MIP-2 expression in keratinocytes is an immediate and early pro-inflammatory response resulting in the recruitment of phagocytic immune cells to clear infections^18–20^. Therefore, we analyzed the composition as well as the potential recruitment of immune cells into the skin of C57BL/6 WT, MyD88-ko, and 5xTLR-ko mice 24 h after epicutaneous treatment with lugdunin or PBS (Fig. 3a–c and Supplementary Fig. 3). In line with the lugdunin-induced MIP-2 and IL-1α induction, we found significantly enhanced recruitment of monocytes and neutrophils in the skin of mice treated with lugdunin, which was completely impaired in MyD88-ko and 5xTLR-ko mice (Fig. 3d). Percentages of live CD45^+^ cells (Fig. 3b), B, T, and NK cells, as well as total CD11b^+^ cells and dendritic cells, were not substantially different compared to the PBS control treatment (Fig. 3c and Supplementary Fig 3). Interestingly, macrophage levels were slightly reduced in percentage upon lugdunin treatment (Fig. 3c and Supplementary Fig. 3). Additionally, we performed immunohistochemical stainings of myeloperoxidase (MPO) in mouse skin sections demonstrating that epicutaneous application of both lugdunin and the lugdunin-producing *S. lugdunensis* results in recruitment of MPO-positive cells into the dermis (Fig. 3e). Taken together, these results indicate that CXCL8/MIP-2 induction in mouse skin and in PHKs is mediated by a TLR/MyD88-dependent pathway in keratinocytes, which leads to the recruitment of phagocytic innate immune cells such as monocytes and neutrophils.

### Lugdunin amplifies innate immune responses of keratinocytes

**Fig. 4:**
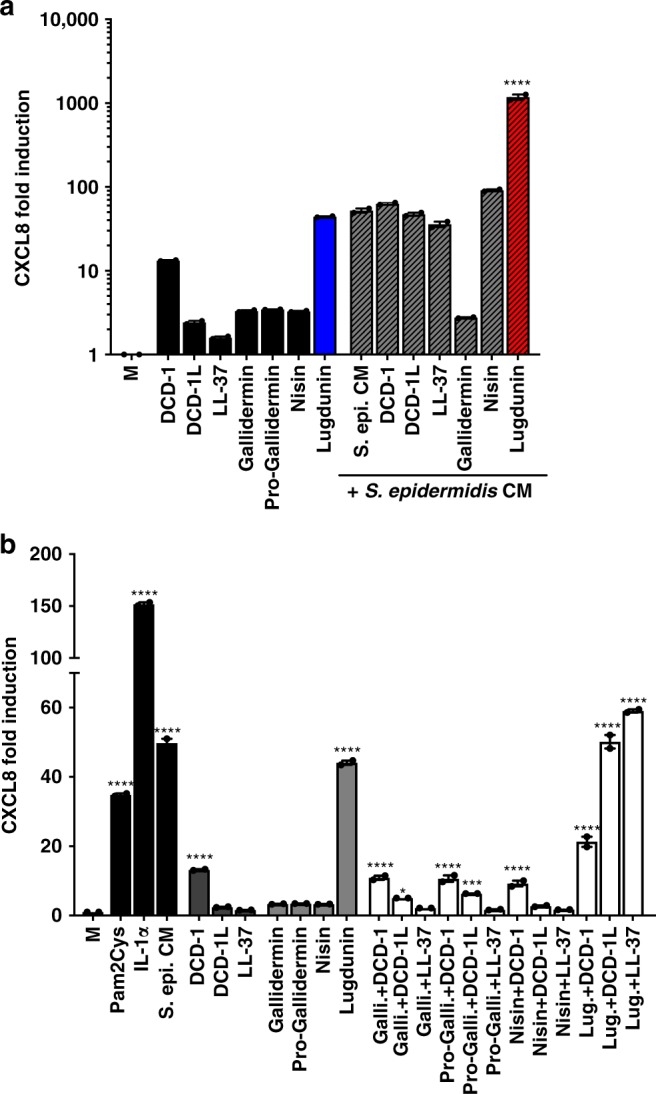
Lugdunin amplifies the commensal-induced chemokine (C-X-C motif) ligand (CXCL8) induction. **a** Primary human keratinocytes (PHKs) were either treated with 2 μM of human antimicrobial peptides (AMPs) (black bars) or lugdunin (blue bars), or 0.8 μM (pro)-gallidermin or nisin (black bars), or in combination with *S. epidermidis* conditioned medium (CM) (gray and red striped bars) for 5 h, and subsequently expression of CXCL8 was analyzed and normalized to actin. Shown is one representative experiment of three independent experiments with two technical replicates ± s.e.m. **b** PHKs were treated with 2 μM human AMPs, 2 μM lugdunin, 0.8 μM of the other bacteriocins (gray bars) or the correspondent peptide combinations (white bars), or 50 ng/mL Pam2Cys, 10 ng/mL IL-1α, or *S. epidermidis* CM as controls (black bars), for 5 h and subsequently expression of CXCL8 was analyzed and normalized to actin. Shown is one representative experiment of three independent experiments with two technical replicates ± s.e.m. Significant differences to control treatments were analyzed by ordinary one-way analysis of variance (ANOVA) followed by Dunnett’s multiple comparisons test (***P < 0.001; ****P < 0.0001). Source data are provided as a Source Data file

Since we showed that lugdunin is a very potent inducer of CXCL8 and AMPs in PHKs, we asked whether other bacteria- and skin-derived AMPs are equally well able to induce CXCL8 expression in PHKs. We tested the bacteriocins nisin and gallidermin, as well as pro-gallidermin, the non-bactericidal pro-form of gallidermin, and the human AMPs LL-37 and the dermcidin-derived peptides DCD-1 and DCD-1L, both of which are secreted by eccrine sweat glands and are thus constitutively present on human skin^21,22^ (Table 1). As shown in Fig. 4a, compared to the other AMPs and bacteriocins, lugdunin was especially potent in inducing CXCL8 expression in PHKs leading to over 40-fold induction. Only DCD-1 treatment resulted in a 10-fold induction of CXCL8 expression in PHKs. LL-37, nisin, and (pro)-gallidermin, however, were not able to induce CXCL8 expression in PHKs. *Staphylococcus epidermidis* CM was equally well able to induce CXCL8 expression in PHKs as lugdunin. Interestingly, CXCL8 induction by *S. epidermidis* CM could be highly amplified by the addition of lugdunin from 40-fold to over 1000-fold (Fig. 4a). This effect was specific for lugdunin since all other AMPs/bacteriocins, except for nisin, which led to a non-significant increase in CXCL8 induction, did not amplify *S. epidermidis*-induced upregulation of CXCL8 expression in PHKs. Surprisingly, gallidermin completely blocked *S. epidermidis* CM-induced CXCL8 expression in PHKs (Fig. 4a).

**Table 1:** Overview of bacteriocins and human AMPs used in this study

Peptide	Sequence	Source	Mode of action
Lugdunin	CvWlVvV (cyclic thiazolidine)	*Staphylococcus lugdunensis*	Unknown
Gallidermin	IASKFLCTPGCAKTGSFNSYCC	*Staphylococcus gallinarum*	Inhibition of cell wall biosynthesis
Pro-gallidermin	MEAVKEKNELFDLDVKVNAKESNDSGAEPRIASKFLCTPGCAKTGSFNSYCC	*Staphylococcus gallinarum*	Inactive pro-form
Nisin	ITSISLCTPGCKTGALMGCNMKTATCHCSIHVSK	*Lactococcus lactis*	Pore formation, inhibition of cell wall biosynthesis
DCD-1	SSLLEKGLDGAKKAVGGLGKLGKDAVEDLESVGKGAVHDVKDVLDSV	Eccrine sweat glands	Pore formation
DCD-1L	SSLLEKGLDGAKKAVGGLGKLGKDAVEDLESVGKGAVHDVKDVLDSVL	Eccrine sweat glands	Pore formation
LL-37	LLGDFFRKSKEKIGKEFKRIVQRIKDFLRNLVPRTES	Epithelial cells, leukocytes	Pore formation

AMPs, antimicrobial peptides

Since lugdunin induced the expression of host-derived AMPs, especially LL-37 in PHKs (Fig. 1c), we asked whether combinations of lugdunin or the other bacterial antimicrobials with LL-37 or the dermcidin-derived peptides DCD-1L and DCD-1 are able to amplify CXCL8 expression in PHKs. Pam2Cys, IL-1α, or *S. epidermidis* CM treatment served as controls. None of the tested peptides exerted cytotoxicity on host cells (Supplementary Fig. 4). As shown in Fig. 4b, lugdunin treatment alone was already very potent in inducing CXCL8 expression in PHKs and the induction level was not significantly amplified further by combined treatments (Fig. 4b). These data indicate that lugdunin is a very potent immune modulator of the skin that acts in concert with other microbiota-derived modulating factors.

### Lugdunin acts synergistically with host-derived AMPs

**Fig. 5:**
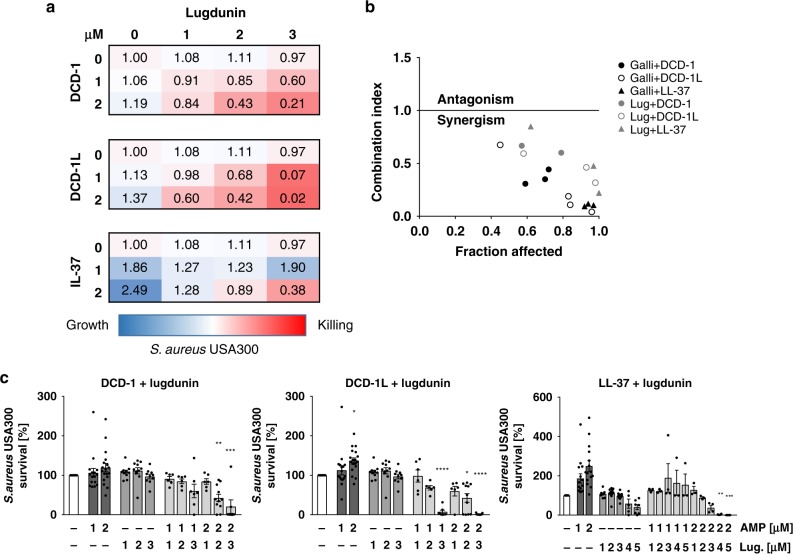
Synergistic action of lugdunin and human antimicrobial peptides (AMPs) kills methicillin-resistant *S. aureus* (MRSA). **a**, **c** Logarithmically grown (3 × 10^6^) *S. aureus* were incubated with indicated combinations of lugdunin and human AMPs in phosphate-buffered saline (PBS) containing 0.1% tryptic soy broth (TSB) at 37 °C orbital shaking (white bar, untreated; dark gray bars, AMP treatment; middle gray bars, lugdunin treatment; light gray bars, AMP and lugdunin combination treatment). After 3 h of incubation, several dilutions of the bacterial suspensions were plated onto TSB agar plates and incubated overnight at 37 °C. The next day *S. aureus* colony-forming units (CFUs) were counted. Each experiment was performed in triplicates. Data represent the percentage of CFU of at least three independent experiments normalized to the untreated control ± s.e.m. Significant differences to control treatments were analyzed by ordinary one-way analysis of variance (ANOVA) followed by Dunnett’s multiple comparisons test (*P < 0.05; **P < 0.01; ***P < 0.001; ****P < 0.0001). **b** Combination indices (CIs) were calculated using CompuSyn (ComboSyn Inc.) and indicated in median effect plots as a function of the bacteria fractions affected by the combinatorial peptide treatment. CI values of 1 indicate additive effects, whereas values <1 and >1 indicate synergistic and antagonistic effects, respectively. Source data are provided as a Source Data file

**Fig. 6:**
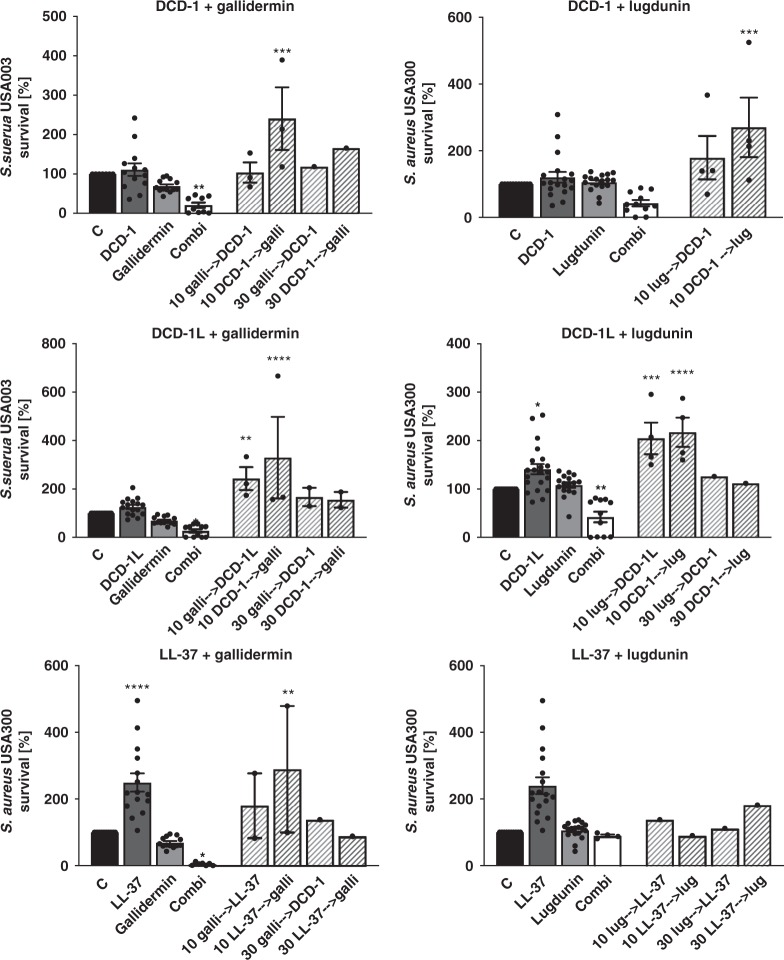
Simultaneous treatment of peptides exhibits antimicrobial activity. Logarithmically grown (3 × 10^6^) *S. aureus* were simultaneously (filled bars; combination treatment (Combi), white bars) or sequentially (striped bars) incubated with 2 µM of human antimicrobial peptides (AMPs) (dark gray bars), 2 µM lugdunin, and 0.8 µM gallidermin (both light gray bars) diluted in phosphate-buffered saline (PBS) containing 0.1% tryptic soy broth (TSB) in a 96-well V-plate. After 10 or 30 min incubation with the first single peptide at 37 °C and orbital shaking, bacteria were collected via centrifugation and were resuspended in a dilution containing the second peptide for 2 h and 50 min, or 2 h and 30 min, respectively. Several dilutions of the bacterial suspensions were plated onto TSB agar plates and incubated overnight at 37 °C. The next day *S. aureus* CFUs were counted. Each experiment was performed in triplicates. Data represent the percentage of CFU normalized to the untreated control ± s.e.m. Significant differences to control treatments were analyzed by ordinary one-way analysis of variance (ANOVA) followed by Dunnett’s multiple comparisons test (*P < 0.05; **P < 0.01; ***P < 0.001; ****P < 0.0001). Source data are provided as a Source Data file

Finally, we analyzed the direct bactericidal effect of lugdunin and the bacteriocins gallidermin and nisin against *S. aureus* and asked whether lugdunin exerts antimicrobial activity in synergy with host-derived AMPs such as DCD-1L, DCD-1, and LL-37. First, we determined sub-bactericidal concentrations of the bacteriocins and AMPs listed in Table 1 against *S. aureus* USA300. The results are shown in Supplementary Fig. 5. We tested combinations of sub-bactericidal concentrations of those bacteriocins/AMPs and analyzed the activity of single or combined treatments of the methicillin-resistant *S. aureus* (MRSA) strain USA300 with these peptides. As shown in Fig. 5a–c and Supplementary Fig. 6, combinations of the human AMPs DCD-1(L) and LL-37 with sub-bactericidal concentrations of lugdunin or gallidermin and nisin resulted in enhanced *S. aureus* killing compared to the single treatments. Of note, the effect was specific for the active form of gallidermin since co-incubation with pro-gallidermin did not lead to antimicrobial activity (Supplementary Figs. 5, 6). Using CompuSyn software, we analyzed potential synergistic effects of peptide combinations and calculated the combination indices for the indicated combinations. The activities of lugdunin and gallidermin in combination with the human AMPs reached combination index (CI) values below 1 indicating synergistic activity of these peptides (Fig. 5b). To analyze whether the synergistic activity is a specific effect on *S. aureus*, we tested the spectrum of antimicrobial activity of lugdunin alone or in combination with DCD-1(L). Supplementary Figure 7 shows that the combined effect of lugdunin with DCD-1(L) could neither be observed for the Gram-positive skin commensal *S. epidermidis* or intestinal *Enterococcus faecalis* nor for the Gram-negative bacteria *Pseudomonas aeruginosa*, *Escherichia coli*, or *Proteus mirabilis*. Of note, *Bacillus subtilis* was the only bacterial species tested besides *S. aureus* that was also susceptible to lugdunin and lugdunin/DCD-1(L) combinations (Supplementary Fig. 7). Additionally, we investigated whether the synergistic activity relies on the combined action of the peptides or whether one peptide is able to sensitize for bacterial killing by the other peptide. Therefore, we performed sequential incubation steps of the single peptides (Fig. 6). Single as well as combination treatments were always included as controls. Interestingly, sequential treatment of *S. aureus* with the synergistically active peptide concentrations did not lead to *S. aureus* killing (Fig. 6). From these data, we conclude that there is a synergistic activity of host- and bacteria-derived peptides in *S. aureus* clearance and that this synergistic effect seems to be dependent on a simultaneous action of the bioactive peptides.

## Discussion

**Fig. 7:**
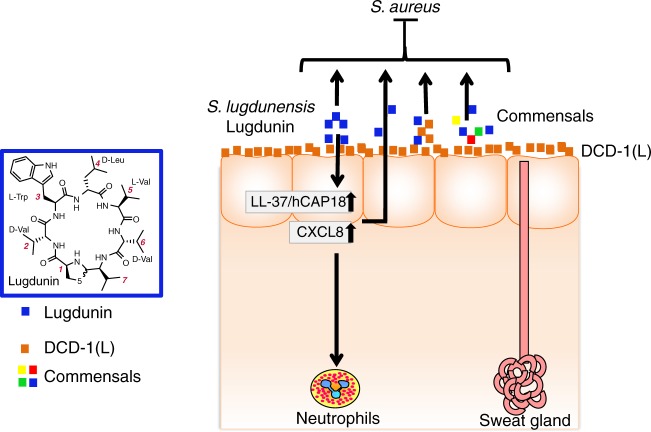
Proposed model of lugdunin-mediated skin protection. Lugdunin acts on different levels to protect against *S. aureus* skin infection: First it can directly inhibit and kill *S. aureus*. Secondly, it can cooperate with host-derived antimicrobial peptides (AMPs) such as hCAP18/LL-37 and the dermcidin-derived peptides DCD-1(L) to kill *S. aureus*. Additionally, on a third level of protection, lugdunin induces an innate immune response of the skin, which leads to the recruitment of phagocytic immune cells, which will clear potentially invading pathogens. Finally, this innate immune response can be highly amplified by factors derived from the skin commensal *S. epidermidis*. Blue, lugdunin; orange, DCD-1(L); multicolored, commensals

Lugdunin was recently discovered by our groups as a novel cyclic peptide antibiotic produced by *S. lugdunensis* that inhibits *S. aureus* nasal and skin colonization in humans and rodent models^10^. In this work, we show for the first time that lugdunin has both immuno-modulatory and bactericidal activities, both of which can be amplified by the presence of other microbiota- or host-derived factors (Fig. 7). Lugdunin particularly induced the expression of the AMP LL-37 and the pro-inflammatory chemokines CXCL8/ MIP-2 in human keratinocytes and mouse skin by a TLR/MyD88-dependent mechanism, which ultimately resulted in the recruitment of neutrophils and monocytes. Furthermore, we show that lugdunin has a synergistic antimicrobial activity against *S. aureus* in combination with skin-derived AMPs. Our results indicate that lugdunin is a multi-functional peptide protecting the host by its direct anti-bacterial activities as well as by sensitizing epithelial cells for increased defense resulting in efficient protection against *S. aureus* skin colonization.

*Staphylococcus lugdunensis* is most frequently found in humans in the inguinal and perineal areas, the axilla, as well as in the nail bed and the nose^10,23^, where it is considered a part of the normal human skin flora. *Staphylococcus lugdunensis* can co-exist with other commensals on human skin such as *S. epidermidis*^23,24^, but intriguingly, nasal colonization by *S. aureus* or *S. lugdunensis*^10,23^ was shown to be mutually exclusive as a result of lugdunin production^10^. In fact, humans who are colonized by *S. lugdunensis* in the nose have a 6-fold lower risk of *S. aureus* carriage than individuals who are not colonized^10^. However, the total number of *S. lugdunensis* in the noses of carriers is considerably low compared to the number of other commensals^10^. Therefore, we speculated that apart from its antimicrobial activity, lugdunin might exhibit additional properties that contribute to the prevention of *S. aureus* colonization. Here we show that the protective potential of lugdunin can be further increased both by combined action with factors produced by other commensals that promote the host innate defense by inducing the expression of AMPs and by recruiting phagocytic immune cells, as well as by synergistic antimicrobial action with the host AMPs LL-37 and dermcidin-derived peptides. These results may explain why such low numbers of *S. lugdunensis* can completely prevent *S. aureus* from colonizing epithelial tissues.

Bacteria from the human microbiota have been found to produce bacteriocins acting against closely related bacteria^25,26^. Lugdunin represents the founding member of the new class of cyclic thiazolidine-containing peptide antibiotics^10^. It exhibits high antimicrobial activity in the micromolar range against a wide range of Gram-positive bacteria, including MRSA, vancomycin-resistant *Enterococcus* isolates, and *Bacillus subtilis* demonstrating its high potency. In our previous work, we have shown that lugdunin treatment led to a strong reduction and even complete eradication of viable *S. aureus* on the surface and in the deeper layers of the skin of mice, demonstrating that lugdunin effectively eradicates *S. aureus* and penetrates tissues in vivo. While lugdunin did neither cause lysis of primary human neutrophils, erythrocytes, or of the human monocytic cell line HL60^10^ nor displayed cell cytotoxicity on PHKs, HNEpCs, HTEpCs, or PBMCS, bacterial cells exposed to lugdunin stopped incorporating radioactive DNA, RNA, protein, or cell wall precursors even at concentrations below the minimal inhibitory concentration, suggesting that lugdunin may lead to a rapid breakdown of bacterial energy resources^10^. Thus, lugdunin can potentially act in concert with other antimicrobial substances to increase bacterial killing. In fact, here we show that lugdunin can enhance the bactericidal activity of host-derived AMPs such as LL-37 or the dermcidin-derived peptides DCD-1(L). While expression of the former can be increased by lugdunin, the latter is constantly present on human skin. Therefore, we suggest that the presence of *S. lugdunensis* on defined skin areas not only increases LL-37 expression in skin but also acts in concert with constitutively expressed host-derived AMPs and by this prevents *S. aureus* skin colonization. Moreover, the synergistic antimicrobial activity of lugdunin with host-derived AMPs seems to be mediated by a combined action since sequential incubation of *S. aureus* with these peptides has no bactericidal effect. We could speculate that lugdunin may act in a similar way as the phenol soluble modulins (PSMs) produced by *S. epidermidis*, which can bind to host-derived AMPs such as LL-37, HBD-2, and HBD-3, and thereby act in a cooperative way to kill *S. aureus*^27^. So far, it is completely unknown whether other peptide antibiotics are equally well able to induce the expression of AMPs in skin. Such synergies of host peptides with peptides derived from skin commensals have a great clinical potential: The group of Richard Gallo recently demonstrated that re-establishment of specific bacteriocin-producing skin commensals in AD patients lacking these commensals prevents *S. aureus* from colonizing the skin of these patients. In this work, they identified novel skin commensal-derived bacteriocins that act synergistically with LL-37. Additionally, a single application of these bacteriocin-producing strains significantly reduced *S. aureus* loads on the forearms of AD patients already 24 h after application^8^.

It has to be determined how lugdunin production is regulated and whether factors from the host side are able to increase lugdunin production by *S. lugdunensis*. This will become especially important when considering the fact that commensals rarely express bacteriocins^10,28^, but expression is induced under habitat-specific stress conditions^28,29^. Thus, we assume that staphylococci as commensals express a basal level of bacteriocins dependent on the habitat and upon entry of a pathogen to the microbial community, bacteriocin expression is further induced resulting in effective host defense.

Besides their bactericidal activity, host-derived AMPs have been shown to play a role in modulation of the innate immune defense^30^ and their expression was shown to be dysregulated in AD patients^30–34^. Dermcidin and its proteolytically processed antimicrobially active peptides DCD-1(L), on the other hand, are constitutively expressed in eccrine sweat glands where they can be transported to the skin surface and serve as a constant antimicrobial shield^21,22^. Thus, being constantly present on the skin surface, our results suggest that DCD-1(L) is able to increase the innate immune defense on skin depending on the available commensal or pathogen-derived bacteriocins. Furthermore, we found that DCD-1(L) alone or in combination with bacteriocins induced CXCL8 expression in keratinocytes. These results are in line with Niyonsaba et al.^35^ who showed that DCD-1L activates nuclear factor-κB (NF-κB) signaling in human keratinocytes and leads to the release of TNFα, CXCL8, interferon-inducible protein 10 (CXCL10), and macrophage inflammatory protein-3α (CCL20)^35^. Additionally, it was recently shown that dermcidin treatment of PBMCs induces the release of TNFα^36^.

By contrast, another study showed that gallidermin was able to totally abolish staphylococcal-induced release of CXCL8 and IL-6 in dermal fibroblasts^37^. Strikingly, these data are in line with our results where gallidermin was able to suppress *S. epidermidis*-induced CXCL8 induction in PHKs. The mechanism still has to be elucidated, but it already demonstrates the potential of defined bacteriocins to counteract harmful inflammatory responses.

Keratinocytes express several pattern recognition receptors such as TLR2, which recognizes *S. aureus* lipopeptides^38^. Activation of TLR2 leads to MyD88-dependent activation of NF-κB and other transcription factors, which subsequently induce the transcription of pro-inflammatory chemokines and cytokines, such as CXCL8 and IL-1α, as well as AMPs involved in cutaneous host defense against *S. aureus*^38^. CXCL8 is a chemokine that recruits neutrophils to the site of infection, whereas IL-1α was shown to be induced in the skin by commensals where it substantially contributes to skin immunity^39,40^. Our novel finding that lugdunin, but not gallidermin or nisin, can induce the secretion of CXCL8 in PHKs and IL-1α in HNEpCs as well as MIP-2 and IL-1α in mouse skin made us speculate that lugdunin has fascinating novel properties for a bacterial peptide and can modulate host cells in a yet mysterious way. More importantly, we could find that lugdunin increases CXCL8 expression in PHKs even further in combination with other commensal-derived factors from *S. epidermidis*.

By analyzing the mechanism of lugdunin-induced CXCL8/MIP-2 induction in skin, we found that lugdunin induces CXCL8/MIP-2 by a TLR/MyD88-dependent mechanism. In MyD88-ko and 5xTLR-ko mice, MIP-2 production induced by lugdunin is impaired in contrast to IL-1α production, which is not affected. TLR2 might play a dominant role since in TLR2-expressing HEK cells lugdunin treatment results in CXCL8 expression and release. Induction levels were, however, substantially lower compared to Pam2Cys/Pam3Cys-mediated TLR2 activation, suggesting that lugdunin might be a weak TLR2 agonist or it might induce CXCL8 expression by an indirect TLR2-activating mechanism. The latter could resemble the mechanism of staphylococcal PSMs, which were shown to mobilize TLR2-activating lipopeptides^16^. Interestingly, however, antimicrobial activity of lugdunin with its original thiazolidine heterocycle building block seems to be crucial for innate immune activation in keratinocytes since the inactive *N*-acetyl lugdunin did not induce CXCL8 expression.

Binding of CXCL8/MIP-2 to CXCR1/2 on neutrophils results in the rapid recruitment of these effector cells to the site of infection^18–20^. In fact, we found that application of lugdunin onto mouse skin results in the recruitment of neutrophils and monocytes only 24 h after topical application. Therefore, clearance of pathogens by lugdunin-mediated recruitment of phagocytic cells complements its direct antimicrobial effects and thus provides an additional level of pathogen protection. The fact that a bacterial cyclic peptide can induce pro-inflammatory chemokine and AMP expression in epithelial cells as well as recruit immune cells to the skin is new and the detailed mechanisms still have to be elucidated in future experiments.

In summary, the results of this study show that lugdunin provides multi-level protection of the host against *S. aureus* (Fig. 7). First, it can act synergistically with the human AMPs DCD-1(L) and LL-37 in killing MRSA. Secondly, lugdunin can amplify the commensal-induced innate immune response in PHKs. And last but not least, lugdunin-induced recruitment of phagocytic cells might additionally contribute to effective eradication of *S. aureus*. It has to be determined whether peptide antibiotics can be used to treat *S. aureus* skin infections in AD patients, but bacterial peptides and human AMP combination therapy may be a new option to combat MRSA skin infections through synergistic antimicrobial effects as well as enhancement of integral pathways of the cutaneous innate immune defense.

## Methods

### Bacterial strains, cells, and culture conditions

The Staphylococci used in this study were *S. aureus* USA300 LAC, *S. epidermidis* 1457, and *S. lugdunensis* IVK28 HR96. *Staphylococcus aureus* and *S. epidermidis* were aerobically grown in tryptic soy broth (TSB) and *S. lugdunensis* in basal medium (BM) at 37 °C and orbital shaking. The antimicrobial testing (AMT) assays were performed with logarithmically growing (optical density (OD) = 0.5) bacteria. Other bacterial strains used in this study were: *Pseudomonas aeruginosa* ATCC27853, *Proteus mirabilis* ATCC29906, *Escherichia coli* ATCC25922, *Enterococcus faecalis* ATCC19434, and *Bacillus subtilis* DB104. *Staphylococcus epidermidis* CM was generated by inoculating 25 mL keratinocyte CnT base medium (CELLnTEC) with 50 µL of an overnight *S. epidermidis* 1457 culture. After 18 h at 37 °C and orbital shaking (OD_600_ = 3), the culture was centrifuged and filter sterilized. Undiluted *S. epidermidis* CM was used in the following experiments.

### Antimicrobial peptides

LL-37 and DCD-1/DCD-1L peptides were synthesized using Fmoc (9-fluorenylmethoxy carbonyl)/tBu chemistry with a multiple peptide synthesizer Syro II (MultiSynTech). After cleavage, peptides were purified by high-performance liquid chromatography (HPLC) on a reversed-phase C18 Nucleosil 100-5C column to a purity of >95% using a linear gradient of 5–80% acetonitrile in 0.05% trifluoroacetic acid for 45 min. Peptides were characterized by matrix-assisted laser desorption ionization-time of flight-mass spectroscopy and electrospray ionization and were in all cases in agreement with the calculated masses.^31,41^. Nisin was purchased from Sigma (#N5764). Gallidermin and pro-gallidermin were isolated from a *Staphylococcus gallinarum* (F16/P57) Tu3928 culture by HCl extraction and reverse-phase HPLC purification^42^. Lugdunin was purified from a *S. lugdunensis* IVK28 culture by 1-butanol extraction, various washing steps, and preparative HPLC^10^. Additionally, lugdunin was synthesized by an Fmoc strategy-based manual solid-phase peptide synthesis^10^. *N*-acetylation of the thiazolidine heterocycle in lugdunin was achieved as follows: typically, 1 mg of lugdunin (1.3 µmol) was dissolved in 200 µL dimethyl sulfoxide. Approximately 100 equivalents of anhydrous sodium carbonate and 1.2 mL of acetic acid anhydride were added and the reaction mixture stirred at room temperature for 24 h. The reaction was quenched by the addition of excess H_2_O. The crude reaction product was purified by standard preparative reversed-phase HPLC and afforded the product *N*-acetyl-lugdunin as a white solid in quantitative yields.

### Antimicrobial testing

For bactericidal testing, logarithmically growing staphylococci were resuspended in PBS (Sigma) containing 0.1% TSB (Carl Roth) and colony-forming unit (CFU) was adjusted to 3 × 10^6^ CFU/mL. Different concentrations of single peptides and their combinations were diluted in PBS containing 0.1% TSB and incubated with bacteria in triplicates for 3 h at 37 °C and 150 rpm orbital shaking. Subsequently, serial dilutions (10^–1^–10^–4^) of the bacterial suspensions were prepared in PBS and 20 µL of each dilution was spotted in duplicates onto TSB plates and incubated at 37 °C overnight. The next day, the number of CFU was analyzed and the percentage of viable bacteria was determined by normalizing to the untreated control (100%). Results are illustrated in a *S. aureus* killing curve. In each experiment, negative control replicates (PBS + 0.1% TSB) as well as sterility control replicates were included.

### Sequential incubation of peptides

Logarithmically growing staphylococci were resuspended in PBS containing 0.1% TSB and CFU was adjusted to 3 × 10^6^ CFU/mL. For sequential peptide incubation, the bacterial suspension was incubated with 2 µM of AMPs, 2 µM lugdunin, or 0.8 µM gallidermin diluted in PBS containing 0.1% TSB in a 96-well V-plate. After 10 or 30 min incubation with the first single peptide at 37 °C and orbital shaking, bacteria were collected via centrifugation for 5 min at 2000 rpm and bacteria were resuspended in a dilution containing the second peptide for 2 h and 50 min or 2 h and 30 min, respectively. The following steps were performed according to the AMT assay described above. In each experiment, combinations of the peptides and the single peptides in the respective concentrations were included as controls as well as sterility control replicates (PBS + 0.1% TSB).

### Cell culture

PHKs and fibroblasts were isolated from human foreskin after routine circumcision from the Loretto Clinic in Tübingen upon informed consent of patients^6,43,44^. Keratinocyte and fibroblast isolation from human foreskin was approved by the ethics committee of the medical faculty of the University Tübingen (654/2014BO2) and performed according to the principles of the Declaration of Helsinki.

After removal of surplus fatty and vascular tissue, the foreskin was cut into small 1 cm^2^ pieces and incubated overnight at 4 °C in epidermal keratinocyte medium with supplements (CELLnTEC) with 10 μg/mL gentamicin and 0.25 μg/mL amphotericin B (CELLnTEC) containing 10 mg/mL Dispase II (Roche) to digest the basal lamina. The next day, epidermis and dermis were carefully separated and small slices of the epidermis were incubated in 0.05% trypsin-EDTA (Merck Millipore) for 30 min, while small slices of the dermis were incubated in 1 mg/mL collagenase A (Roche) in fibroblast medium (CELLnTEC). Digestion was stopped using Roswell Park Memorial Institute (RPMI)-1640 medium (Thermo Fisher Scientific) containing 10% fetal bovine serum (FBS, Biochrom), and single cells were obtained using a 100-μm-pore-size cell strainer (Corning Incorporated). After centrifugation, cells were resuspended in epidermal keratinocyte medium with supplements (CELLnTEC) or fibroblast medium (CELLnTEC), respectively.

PHKs were cultured in collagen-coated tissue flasks (Corning, BioCoat^TM^) in epidermal keratinocyte medium (CELLnTEC) at 37 °C, 5% CO_2_^6,43,44^. Twenty-four hours prior to experiments, keratinocytes were differentiated with 1.7 mM CaCl_2_ in epidermal keratinocyte base medium (CELLnTEC). Primary human fibroblasts were cultured in fibroblast medium (CELLnTEC). Primary human tracheal (HTEpCs) and nasal epithelial cells (HNEpCs) (PromoCell) were kindly provided by J. Schade (Interfaculty Institute of Microbiology and Infection Medicine Tübingen) and cultured in airway epithelial cell growth medium (PromoCell). HEK293 cells were cultured in Dulbecco’s modified Eagle’s medium (Sigma) supplemented with 10% FBS (Biochrom). For HEK293-TLR2 cells, 10 µg/mL normocin (InvivoGen) and 10 µg/mL blasticin (InvivoGen) were added to the culture medium. Before peptide stimulation fresh medium was added to cells. HEK293 and HEK293-TLR2 cells (InvivoGen) were kindly provided by D. Kretschmer (Interfaculty Institute of Microbiology and Infection Medicine Tübingen).

### 3D human skin equivalent

For 3D human skin equivalents, 1.35 mg/mL neutralized (pH 7.2–7.4) collagen I (Corning) was diluted in fibroblast medium (CELLnTEC) and 1 mL of collagen solution was added to 6-well inserts (0.4 µM, Merck). After 2 h of incubation at 37 °C, 8 × 10^5^ fibroblasts diluted in 3 mL fibroblast medium were seeded on top of the collagen matrix. Subsequently, fibroblast medium was added to the bottom compartment of the insert. Dermal equivalents were incubated at 37 °C, and on days 2 and 4, fresh fibroblast medium was added. On day 5, 1 × 10^6^ PHKs in 100 µL CnT (CELLnTEC) were seeded on top of the dermis. Concurrently, the medium in the bottom compartment was changed to CnT medium. From then on, the medium was changed every second day until day 12. From then on, skin equivalents were airlifted and the medium in the bottom compartment was changed to airlift medium (CELLnTEC). The medium was changed every second day until day 22.

On day 22, 1.5 µg lugdunin in 10 µL PBS were topically applied for 24 h onto the epidermis by using 8 mm filter paper discs (Smart Practice). The next day, the cell culture supernatant was used for ELISA (enzyme-linked immunosorbent assay)/Legendplex^TM^.

### Adhesion and invasion assay

Adhesion and invasion assays were performed by incubating differentiated PHKs with 2 µM lugdunin, *S. epidermidis*-CM or a combination of both or medium as a control for 18 h. The next day, keratinocyte supernatant was removed, keratinocytes were washed twice with Hank’s balanced salt solution (HBSS) (Sigma), and fresh keratinocyte base medium containing 1.7 mM CaCl_2_ was added. Subsequently, keratinocytes were infected with *S. aureus* (multiplicity of infection = 30; OD = 0.5) for 1.5 h. After two washing steps with HBSS, keratinocytes were lysed and serial dilutions of the lysates were plated onto blood agar plates. After overnight incubation at 37 °C CFUs were counted.

### LEGENDplex^TM^ multiplex cytokine analysis

For cytokine analysis from cell cultures, 10 µL of supernatant was used for cytokine analysis via the LEGENDplex^TM^ human inflammation panel (BioLegend). For cytokine analysis from mouse skin, 4 mm skin punches were cultured in an airlift system where only the dermis had access to medium for 10 h. Skin punches were cultured in RPMI containing 1% FCS (Biochrom/Merck Millipore), 1% penicillin and streptomycin (Gibco/Life Technologies) and 0.25 µg/mL amphotericin B (CELLnTEC). Ten microliters of a 3-fold dilution of culture supernatant was used for cytokine analysis via the LEGENDplex^TM^ mouse inflammation panel (BioLegend). Samples were acquired in duplicates using a BD LSRII flow cytometer (BD Biosciences) and LEGENDplex^TM^ Software (BioLegend).

### Enzyme-linked immunosorbent assay

For IL-1α analysis, 100 µL of cell culture supernatant was analyzed via ELISA (R&D Systems) according to manufacturer’s instruction. For MIP-2 analysis, 100 µL of a 5-fold dilution from skin cultures supernatant was used and analyzed via ELISA (R&D Systems) according to the manufacturer’s instruction. For LL-37 ELISA, ELISA plates (Nunc) were coated overnight at 4 °C with 100 µL of cell culture supernatant or 2-fold dilutions of LL-37 starting from 8 µg/mL. The next day, the plate was washed three times using PBS + 0.5% BSA + 0.05% Triton X-100, followed by incubation with 100 µL primary antibody against hCAP18/LL-37 (HycultBiotech, Cat#HM2071, 1:120 in PBS + 0.5% BSA + 0.05% Triton X-100) at 37 °C for 1 h. After washing, incubation with 100 µL secondary antibody (Cell Signaling, Cat#7076S, 1:3000 in PBS + 0.5% BSA + 0.05% Triton X-100) followed at 37 °C for 1 h. Subsequently, plates were washed and 100 µL TMB substrate solution (Cell Signaling) was added. Reaction was stopped with 50 µL 2 N H_2_SO_4_ and absorbance at 450 nm was measured using a Fluoroskan II (Labsystems).

### Immunohistochemistry

For immunohistochemical stainings of mouse skin, 3 µM tissue sections were de-paraffinized. For MPO staining, antigen retrieval was performed in EDTA buffer at pH 9 (Thermo Fisher Scientific) for 5 min, and for MIP-2 and LL-37, citrate buffer at pH 6 for 9 min was used. Antigen retrieval was performed in a pressure cooker before a slow cooling down of the samples in the buffer. Afterwards, tissue sections were washed in PBS and blocked in 5% donkey serum in PBS containing 0.05% Triton X-100 for 90 min. For MPO staining, tissue sections were subsequently incubated overnight at 4 °C in a humid chamber with a MPO-specific antibody (R&D Systems, Cat#AF3667) diluted 1:50 in a blocking buffer. For MIP-2 and LL-37 staining, tissue sections were first incubated with primary enhancer (Lab Vision™ UltraVision™ LP Detection System, Thermo Fisher Scientific) for 20 min at room temperature, washed, and then incubated with AP polymer (Lab Vision™ UltraVision™ LP Detection System, Thermo Fisher Scientific) for 30 min at room temperature in a humid chamber, followed by incubation with the primary antibodies MIP-2 (Thermo Scientific, Cat#701126, 1:10 in blocking buffer) and LL-37 (Novus Biologicals, Cat#NB100-98689, 1:200 in blocking buffer) overnight at 4 °C in a humid chamber. The next day, tissue sections were washed and incubated with a 1:250 dilution of alkaline phosphatase-coupled secondary antibody (Novus Biologicals) for 90 min in a humid chamber at room temperature. After washing in PBS, staining was performed by using the Lab Vision^TM^ liquid fast red substrate system (Thermo Fisher Scientific) according to the manufacturer’s instructions. After washing in water, hematoxylin–eosin staining (Agilent/Dako) was performed for 2 min. After another washing step in water, tissue sections were mounted with Kaiser’s glycerol gelatine (Merck).

### RNA isolation and cDNA synthesis

After 5 h or 20 h of peptide treatment, PHKs were washed once with PBS, followed by the addition of RNA lysis buffer directly into the well. Total RNA was extracted using the Nucleospin RNA Kit (Macherey-Nagel) according to the manufacturer’s protocol. Complementary DNA was synthesized using the Reverse-Transkriptase Kit (Thermo Scientific) with 2 µg of RNA, 4 µL of 5× RT buffer, 0.5 µL Maxima reverse transcriptase (200 U/mL), 1 µL of random hexamer primer (100 µM), dNTP (10 mM), and RNAse-free water to a total volume of 20 µL. After pre-incubation of RNA with water for 10 min at 70 °C, master mix was added and incubated for 10 min at 25 °C, followed by 45 min at 50 °C and a final heat inactivation step for 5 min at 85 °C.

### Quantitative reverse transcription-polymerase chain reaction

Quantitative reverse transcription-polymerase chain reaction (qRT-PCR) was performed in 10 µL reaction volume with SYBR^TM^ Green PCR Master Mix (Thermo Fisher) according to the manufacturer’s instructions using a LightCycler 96 (Roche Life Science). The initial denaturation step was at 95 °C for 5 min, followed by 40 cycles with 10 s each for the denaturation step at 95 °C, the annealing at individual temperature, and the elongation at 72 °C. Primer sequences and respective annealing temperatures are listed in Supplementary Table 1.

### PBMC isolation

PBMC isolation from human blood was approved by the ethics committee of the medical faculty of the University of Tübingen (054/2017BO2).

Human PBMCs were isolated from the peripheral blood of healthy donors upon obtaining informed consent by Ficoll-Histopaque (Biochrom) gradient centrifugation. Cells were washed once in PBS and adjusted to a cell number of 1 × 10^6^ mL^−1^ in RPMI-1640 medium (Gibco/Life Technologies) containing 10% FBS (Biochrom/Merck Millipore).

### Viability assay

Effects of the used peptides on keratinocytes, PBMCs, and nasal and tracheal epithelial cell viability were tested using 4-methylumbelliferyl heptanoate (MUH). Briefly, cells were treated with peptides and respective peptide combinations for 24 h, followed by incubation with 100 μg/mL MUH (Sigma-Aldrich) in PBS for 1 h at 37 °C. The absolute fluorescence intensity at *λ*_ex_ of 355 nm and *λ*_em_ of 460 nm was measured using a Fluoroskan II (Labsystems).

### Mouse model

All mouse experiments were conducted in accordance with the German regulations of the Gesellschaft für Versuchstierkunde/Society for Laboratory Animal Science (GV-SOLAS) and the European Health Law of the Federation of Laboratory Animal Science Associations (FELASA). All mouse experiments were approved (HT1/12; HT1/17) by the local authorities (Regierungspräsidium Tübingen). Animal studies were performed with 6–8-week-old female C57BL/6 WT/MyD88-ko/5xTLR-ko (TLR2^−/−^; TLR3^−/−^; TLR4^−/−^; TLR7^−/−^; TLR9^−/−^) mice.

Mouse skin was shaved 3 days prior to experiments allowing potential micro wounds to heal and skin to recover from shaving. To analyze *S. aureus* skin colonization, 2 × 15 µL (right and left flank) containing 1.5 µg lugdunin, *S. epidermidis* CM, the combination of both, or PBS were epicutaneously applied for 24 h on the shaved back skin of C57BL/6 WT mice by using 8 mm filter paper discs and Finn Chambers (Smart Practice). The next day, Finn Chambers were removed and 2 × 15 µL of a bacterial suspension containing 1 × 10^8^ *S. aureus* were epicutaneously applied using new filter paper discs and new Finn Chambers. After 24 h, mice were euthanized and 4 mm skin punches were used for *S. aureus* CFU analysis.

To analyze the immune cell composition and cytokines in the skin, 15 µL containing 1.5 μg lugdunin or PBS as a control were epicutaneously applied for 24 h on the shaved back skin of C57BL/6 WT/MYD88-ko/5xTLR-ko mice. After 24 h, mice were euthanized and relevant skin areas or 4 mm skin punches were removed for immune cell analysis and LEGENDplex^TM^ (BioLegend) cytokine analysis.

### Mouse immune cell isolation and staining procedure

To prepare single-cell suspensions, relevant dorsal skin area was transferred to PBS + 2% FBS (Biochrom/Merck Millipore). Subcutaneous fat was removed using a razor blade and skin tissue containing epidermal and dermal parts was transferred into a 2 mL reaction tube containing digestion solution. Digestion solution contained 0.05 mg/mL DNase I (Roche) and 0.25 mg/mL Liberase (Roche) in RPMI-1640 Medium (Gibco/Life technologies). After scissor-mediated tissue disintegration digestion was performed for 1 h at 37 °C and stopped by the addition of 100 µL of FCS (Biochrom/Merck Millipore). Single cells were separated by using an 80 µm cell strainer (Greiner Bio-One). After washing in PBS + 2% FBS, single-cell suspensions were treated with TruStain fcX^TM^ anti-CD16/32 (1:50, BioLegend) and subsequently surface stained with the following monoclonal antibodies: CD45.2 (1:200, BioLegend, clone 104, Cat#109824), F4/80 (1:200, BioLegend, clone BM8, Cat#123110), CD11b (1:200, BioLegend, clone M1/70, Cat#101227), CD11c (1:200, BioLegend, clone N418, Cat#117337), Ly6G (1:200, BioLegend, clone 1A8, Cat#127614), Ly6C (1:200, BioLegend, clone HK1.4, Cat#128014), CD19 (1:200, BioLegend, clone 6D5, Cat#115508), CD3 (1:200, BioLegend, clone 17A2, Cat#100214), and NK1.1 (1:200, BioLegend, clone PK136, Cat#108714). Fixable viability dye eFluor520 (1:1000, eBioscience^TM^) was used to exclude dead cells. All samples were acquired using a BD LSRII flow cytometer (BD Biosciences) and analyzed with FlowJo (Treestar).

### Quantification and statistical analysis

Significant differences between the means of the different treatments were evaluated using GraphPad Prism 7.0 (GraphPad Software, Inc.). Either unpaired, two-tailed Student’s *t* test or one-way analysis of variance followed by Dunnett’s multiple comparisons test was used for statistical analysis and indicated in the respective figure legends. Differences were considered statistically significant with a *p* value of <0.05. To evaluate potential synergistic effects of peptide combinations, the respective CIs were calculated using CompuSyn (ComboSyn Inc.) and indicated in median effect plots as a function of the bacteria fractions affected by the combinatorial peptide treatment. CI values of 1 indicate additive effects, whereas values <1 and >1 indicate synergistic and antagonistic effects, respectively. Data are visualized using GraphPad Prism 7.0 (GraphPad Software Inc.), MS Excel (Microsoft Corporation), or FlowJo (Treestar).

### Reporting summary

Further information on research design is available in the Nature Research Reporting Summary linked to this article.

## Supplementary information

Supplementary Information

Reporting Summary

Source Data

## Data Availability

The authors declare that all the data that support the findings of this study are available from the corresponding author upon request. The source data underlying Figs. 1, 2a–e, 3b, d, 4, 5b, 5c, 6 and Supplementary Figures 1, 2a–c, 3, 4, 5 are provided as a Source Data file.
