# Supplementary material for: Lugdunin amplifies innate immune responses in the skin in synergy with host- and microbiota-derived factors
Source: Nat Commun. 2019 Jun 21;10:2730. doi: 10.1038/s41467-019-10646-7 (PMC6588697; doi:10.1038/s41467-019-10646-7)
Supplement: Supplementary file 1 — Supplementary Information [file 41467_2019_10646_MOESM1_ESM.pdf]

Supplementary Information

Lugdunin amplifies innate immune responses in the skin in synergy with host- and microbiota-derived factors

Bitschar et al.

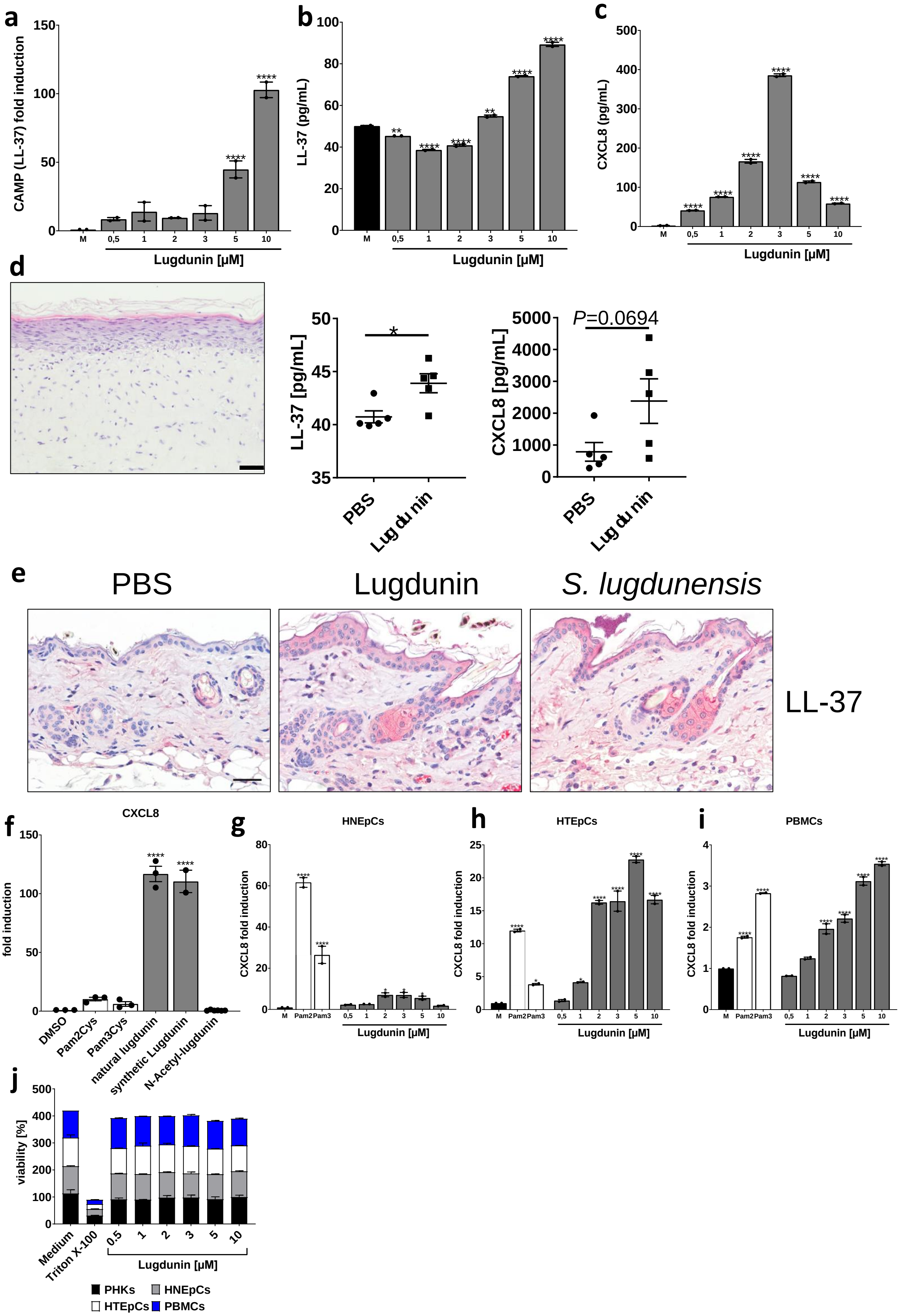

Supplementary Figure 1: Lugdunin-induced LL-37 is concentration-dependent and CXCL8 is cell type-specific

a: PHKs were treated with increasing concentrations of lugdunin for 24 hours and subsequently expression of CAMP (LL-37) was analyzed and normalized to actin. Shown is one representative experiment of three independent experiments with two technical replicates each +/-s.e.m. Black bar: medium control.

b+c: PHKs were treated with increasing concentrations of lugdunin for 24 hours and subsequently the concentration of LL-37 and CXCL8 in the supernatant was analyzed. Shown is one representative experiment of three independent experiments, each with two technical replicates +/-s.e.m.

d: Representative hematoxylin-eosin-stained paraffin-embedded human 3D skin equivalent. Scale bar = 100 µM. 1.5 µg lugdunin or PBS was topically applied onto 3D skin equivalents. After 24 hours, the concentration of LL-37 and CXCL8 in the supernatant was analyzed. One dot represents one skin equivalent. Shown is the mean percentage +/- s.e.m. Significant differences to control treatments were analyzed by an unpaired two-tailed t-test (*P<0.05).

e: Representative LL-37-stained paraffin-embedded mouse skin sections. Scale bar = 100 µM.

f: PHKs were treated with 2 µM natural lugdunin, synthetic lugdunin, N-Acetyl lugdunin (grey bars) or 100 ng/mL Pam2Cys or Pam3Cys (white bars) as positive control for 5 hours and subsequently expression of CXCL8 was analyzed and normalized to actin. Black bar: DMSO control. Shown is one representative experiment of three independent experiments with at least two technical replicates +/-s.e.m.

g+h+i: HNEpCs (**b**), HTEpCs (**c**) or PBMCs (**d**) were treated with increasing concentrations of lugdunin (grey bars) or 100 ng/mL Pam2Cys or Pam3Cys (white bars) as positive controls for 5 hours and subsequently expression of CXCL8 was analyzed and normalized to actin. Black bar: medium control. Shown is one representative experiment of three independent experiments with two technical replicates +/-s.e.m. Significant differences to control treatments were analyzed by ordinary one-way ANOVA followed by Dunnett's multiple comparisons test (*P<0.05; **P<0.01; ***P<0.001; ****P<0.0001).

j: PHKs, HNEpCs, HTEpCs or PBMCs were treated with increasing concentrations of lugdunin or 0.1% Triton X-100 for 24 hours and subsequently incubated with 4-methylumbelliferyl heptanoate. Treatment with 0.1% Triton X-100 was used as negative control. Data were normalized to the untreated control. Shown is one

representative experiment of three independent experiments with two technical replicates +/-s.e.m. Source data are provided as a Source Data file.

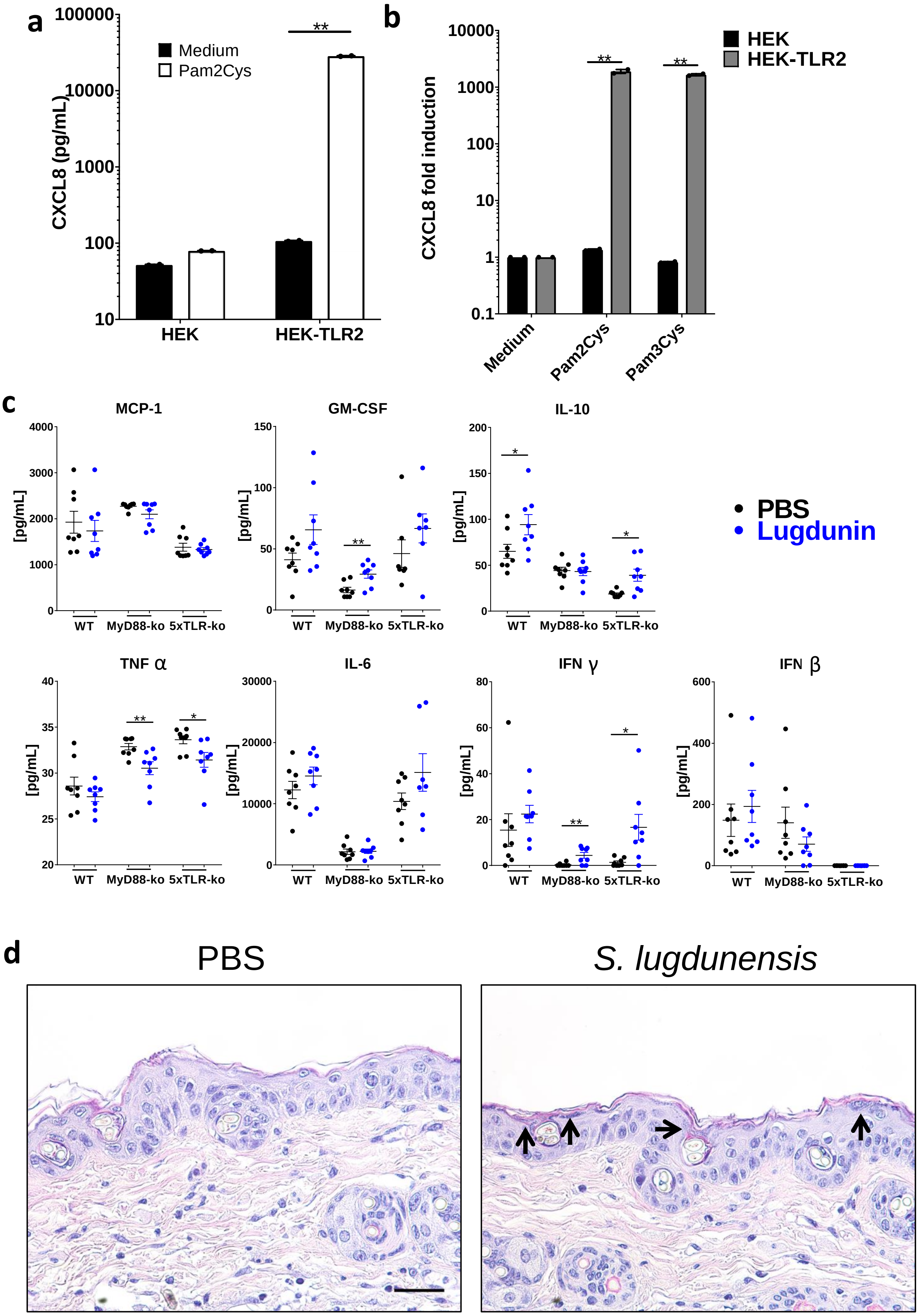

Supplementary Figure 2: CXCL8 controls and other cytokines in mouse skin

a: HEK-control cells or HEK-TLR2 cells were treated with 100 ng/mL Pam2Cys for 5 hours and subsequently the CXCL8 concentration in the supernatant was analyzed.

Shown is one representative experiment of three independent experiments with two technical replicates +/-s.e.m.

b: HEK-control cells or HEK-TLR2 cells were treated with 100 ng/mL Pam2Cys or Pam3Cys for 5 hours and subsequently expression of CXCL8 was analyzed. Shown is one representative experiment of three independent experiments with two technical replicates +/-s.e.m.

c: Shown are the mean concentrations of the indicated cytokines in the supernatant of the organ skin culture of two skin punches from four mice each +/- s.e.m.

Significant differences to control treatments were analyzed by an unpaired two-tailed t-test (*$P<0.05$; **$P<0.01$; ***$P<0.001$; ****$P<0.0001$).

d: Representative hematoxylin-eosin-stained paraffin-embedded mouse skin sections. Scale bar = 100 µM.

Source data are provided as a Source Data file.

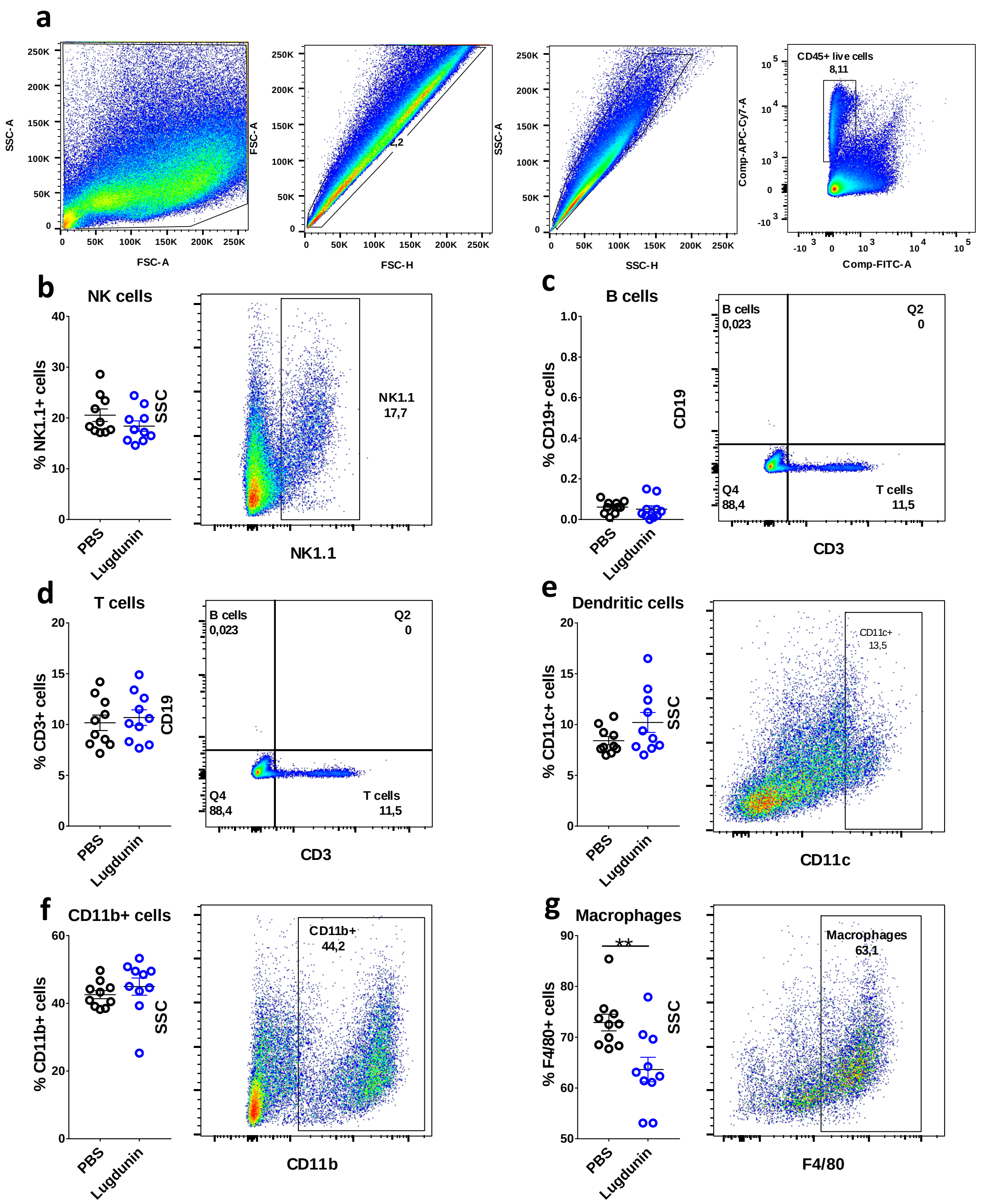

Supplementary Figure 3: Gating strategy for immune cell analysis in mouse skin

a: Gating strategy for live CD45+ cells. Gating for the different immune cell subsets pre-gated on live CD45+ cells: NK cells (**b**); B cells (**c**); T cells (**d**); Dendritic cells (**e**); CD11b+ cells (**f**); Pregated on CD11b+ CD45+ live cells presented on Figure 3d. Macrophages (**g**). Shown is the mean percentage of indicated immune cells in mouse skin of 10 C5BL/6 WT mice +/- s.e.m. One dot represents one mouse.

Source data are provided as a Source Data file.

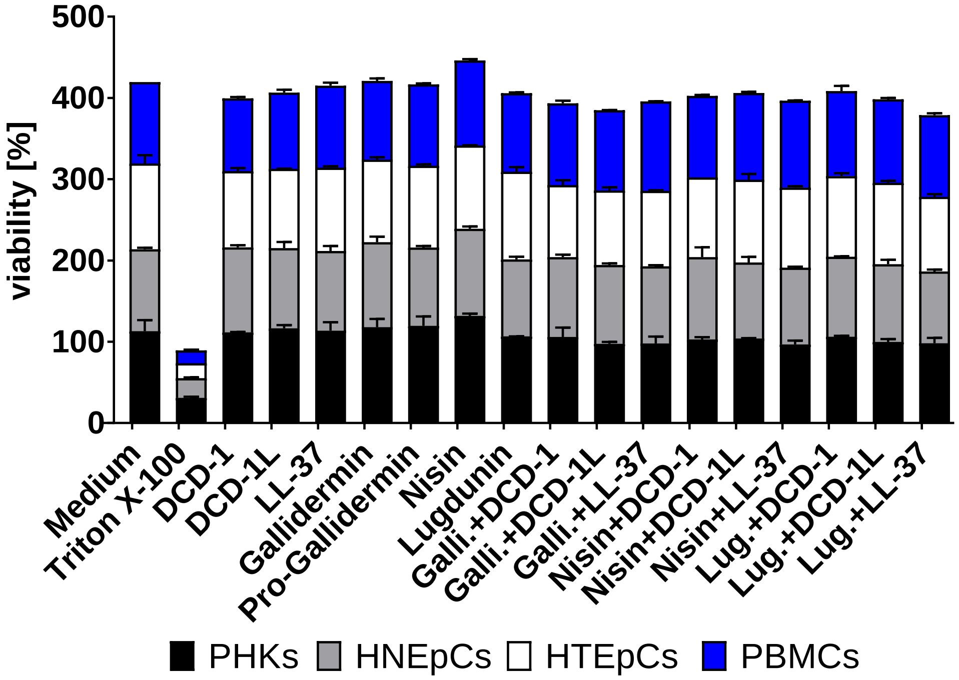

Supplementary Figure 4: Viability of primary cells upon AMP treatment

PHKS, HNEpCs, HTEpCs or PBMCs were treated with 2 μM human AMPs, 2 μM lugdunin, 0.8 μM of indicated bacteriocins or the correspondent peptide combinations for 24 hours. Subsequently, cells were incubated with 4-methylumbelliferyl heptanoate and viability was calculated. Treatment with 0.1 % Triton X-100 was used as negative control. Data were normalized to the untreated control. Shown is one representative experiment of three independent experiments with two technical replicates +/-s.e.m. Source data are provided as a Source Data file.

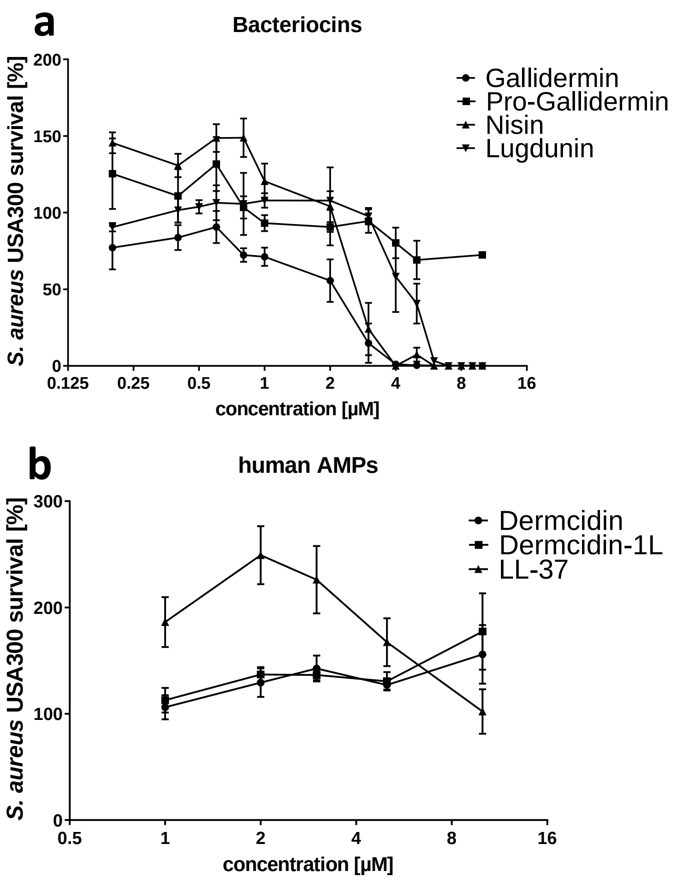

Supplementary Figure 5: Determination of minimal bactericidal concentrations of peptides used against *S. aureus* USA300

a+b: $3x10^6$ logarithmically grown *S. aureus* were incubated with indicated concentrations of the bacteriocins (pro)-gallidermin, nisin and lugdunin (**a**) or the human AMPs Dermcidin-1(L) and LL-37 (**b**) in PBS containing 0.1% TSB at 37 °C orbital shaking. After 3 hours of incubation several dilutions of the bacterial suspensions were plated onto TSB agar plates and incubated over night at 37 °C. The next day *S. aureus* CFU were counted. Each experiment was performed in triplicates. Data represent the mean percentage of *S. aureus* survival measured in CFU and normalized to the untreated control. Data represent the mean of at least three independent experiments +/- s.e.m. Source data are provided as a Source Data file.

a

µM		Gallidermin 0	Gallidermin 0,4	Gallidermin 0,6	Gallidermin 0,8	Pro-Gallidermin 0	Pro-Gallidermin 0,4	Pro-Gallidermin 0,6	Pro-Gallidermin 0,8
DCD-1	0	1,00	0,84	0,91	0,74	1,00	1,34	1,61	1,30
DCD-1	1	1,06	0,61	0,65	0,76	1,06	1,06	1,05	1,48
DCD-1	2	1,19	0,41	0,30	0,28	1,19	1,53	1,47	1,73
DCD-1L	0	1,00	0,84	0,91	0,74	1,00	1,34	1,61	1,30
DCD-1L	1	1,13	0,75	0,67	0,55	1,13	1,57	1,22	1,08
DCD-1L	2	1,37	0,16	0,05	0,17	1,37	1,78	1,47	1,81
LL-37	0	1,00	0,84	0,91	0,74	1,00	1,34	1,61	1,30
LL-37	1	1,86	1,20	1,04	0,56	1,86	1,54	1,61	1,81
LL-37	2	2,49	0,08	0,06	0,03	2,49	2,64	2,03	2,22

b

µM		Nisin 0	Nisin 0,8
DCD-1	0	1,00	1,49
DCD-1	1	1,06	1,08
DCD-1	2	1,19	0,85
DCD-1L	0	1,00	1,49
DCD-1L	1	1,13	1,02
DCD-1L	2	1,37	0,32
LL-37	0	1,00	1,49
LL-37	1	1,86	1,39
LL-37	2	2,49	0,00

c

µM		Lugdunin 0	Lugdunin 1	Lugdunin 2	Lugdunin 3
Gallidermin	0,0	1,00	1,08	1,11	0,97
Gallidermin	0,4	0,84	0,85	0,74	0,89
Gallidermin	0,6	0,91	0,81	0,80	0,87
Gallidermin	0,8	0,74	1,09	0,68	0,81
Nisin	0,0	1,00	1,08	1,11	0,97
Nisin	0,4	1,31	0,96	0,94	0,80
Nisin	0,6	1,49	0,78	0,91	0,79
Nisin	0,8	1,49	0,87	0,88	1,05

d

µM		LL-37 0	LL-37 1	LL-37 2
DCD-1	0	1,00	1,86	2,49
DCD-1	1	1,06	1,90	1,74
DCD-1	2	1,19	1,78	1,96
DCD-1L	0	1,00	1,86	2,49
DCD-1L	1	1,13	2,20	2,26
DCD-1L	2	1,40	2,61	2,08

Growth — Killing
S. aureus USA300

Supplementary Figure 6: Combination treatments of *S. aureus* with different AMPs and bacteriocins

$3x10^6$ logarithmically grown *S. aureus* were incubated with indicated combinations of the bacteriocins (pro)-gallidermin (**a**) or nisin (**b**) with human AMPs or with combinations of lugdunin with gallidermin and nisin (**c**) or with combinations of DCD-1(L) and LL-37 (**d**) in PBS containing 0.1% TSB at 37 °C orbital shaking. After 3 hours of incubation several dilutions of the bacterial suspensions were plated onto TSB agar plates and incubated over night at 37 °C. The next day *S. aureus* CFU were counted. Each experiment was performed in triplicates. Data represent the mean percentage of CFU normalized to the untreated control. Data represent the mean of at least three independent experiments.

a ***S. epidermidis***

	μM	Lugdunin 0	1	2	3
DCD-1	0	1,00	1,08	0,86	1,20
	1	1,51	1,90	1,37	1,04
	2	0,94	1,57	1,18	1,16
DCD-1L	0	1,00	1,08	0,86	1,20
	1	1,43	1,29	1,27	1,08
	2	1,33	1,43	1,06	1,02

b ***P. aeruginosa***

	μM	Lugdunin 0	1	2	3
DCD-1	0	1,00	1,34	1,01	1,41
	1	0,93	1,37	1,49	1,56
	2	1,21	1,73	1,36	1,44
DCD-1L	0	1,00	1,34	1,01	1,41
	1	1,39	1,31	1,31	1,50
	2	1,19	1,16	0,87	0,93

c ***P. mirabilis***

	μM	Lugdunin 0	1	2	3
DCD-1	0	1,00	1,13	0,84	0,98
	1	1,05	1,38	1,05	1,15
	2	0,98	1,03	1,30	1,23
DCD-1L	0	1,00	1,13	0,84	0,98
	1	0,95	1,05	0,74	0,92
	2	1,10	1,31	1,11	0,98

d ***E. coli***

	μM	Lugdunin 0	1	2	3
DCD-1	0	1,00	1,05	1,34	1,27
	1	1,31	0,98	1,40	1,27
	2	1,87	1,52	1,44	1,47
DCD-1L	0	1,00	1,05	1,34	1,27
	1	1,26	1,23	1,31	1,27
	2	1,92	1,34	1,31	1,31

e ***E. faecalis***

	μM	Lugdunin 0	1	2	3
DCD-1	0	1,00	1,21	1,27	0,99
	1	1,48	1,42	1,73	1,48
	2	1,58	1,69	1,30	1,09
DCD-1L	0	1,00	1,21	1,27	0,99
	1	1,40	1,21	1,30	1,39
	2	1,55	1,32	1,25	1,25

f ***B. subtilis***

	μM	Lugdunin 0	1	2	3
DCD-1	0	1,00	0,81	0,60	0,44
	1	1,14	0,53	0,33	0,10
	2	1,04	0,60	0,06	0,03
DCD-1L	0	1,00	0,81	0,60	0,44
	1	0,96	0,65	0,28	0,06
	2	0,63	0,11	0,06	0,03

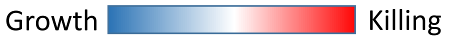

Supplementary Figure 7: Efficiency of Lugdunin and DCD-1(L) combinations in bacteria killing

3x10[6] logarithmically grown bacteria were incubated with indicated combinations of lugdunin and DCD-1(L) in PBS containing 0.1% TSB at 37 °C orbital shaking. After 3 hours of incubation several dilutions of the bacterial suspensions were plated onto TSB agar plates and incubated over night at 37 °C. The next day bacterial CFU were counted. Each experiment was performed in triplicates. Data represent the mean percentage of CFU normalized to the untreated control. Data represent the mean of at least three independent experiments.

Supplementary Table 1: List of Primers used in this study

**Primer**	**Sequence**	**Annealing Temp**
ACTB fw	TTGTTACAGGAAGTCCCTTGCC	60 °C
ACTB rv	ATGCTATCACCTCCCCTGTGTG	
CXCL8 fw	AGACAGCAGAGCACACAAGC	60 °C
CXCL8 rv	ATGGTTCCTTCCGGTGGT	
CAMP fw	TCGGATGCTAACCTCTACCG	58 °C
CAMP rv	GTCTGGGTCCCCATCCAT	
DEFB1 fw	TGTCTGAGATGGCCTCAGGT	60 °C
DEFB1 rv	GGGCAGGCAGAATAGAGACA	
DEFB4A fw	TCAGCCATGAGGGTCTTGTA	58 °C
DEFB4A rv	GGATCGCCTATACCACCAAA	
DEFB103A fw	TTCTGTTTGCTTTGCTCTTCC	62 °C
DEFB103 rv	CGCCTCTGACTCTGCAATAAT	
RNASE7 fw	GAAGACCAAGCGCAAAGC	58 °C
RNASE7 rv	CAGCAGAAGCAGCAGAAGG	
