# Supplementary material for: Lugdunin amplifies innate immune responses in the skin in synergy with host- and microbiota-derived factors
Source: Nat Commun. 2019 Jun 21;10:2730. doi: 10.1038/s41467-019-10646-7 (PMC6588697; doi:10.1038/s41467-019-10646-7)
Supplement: Supplementary file 2 — Reporting Summary [file 41467_2019_10646_MOESM2_ESM.pdf]

nature research

Corresponding author(s): Birgit Schittek (birgit.schittek@med.uni-tuebingen.de)

Reporting Summary

Nature Research wishes to improve the reproducibility of the work that we publish. This form provides structure for consistency and transparency in reporting. For further information on Nature Research policies, see Authors & Referees and the Editorial Policy Checklist.

Statistical parameters

When statistical analyses are reported, confirm that the following items are present in the relevant location (e.g. figure legend, table legend, main text, or Methods section).

n/a	Confirmed	
☐	☒	The exact sample size (*n*) for each experimental group/condition, given as a discrete number and unit of measurement
☐	☒	An indication of whether measurements were taken from distinct samples or whether the same sample was measured repeatedly
☐	☒	The statistical test(s) used AND whether they are one- or two-sided<br>*Only common tests should be described solely by name; describe more complex techniques in the Methods section.*
☒	☐	A description of all covariates tested
☐	☒	A description of any assumptions or corrections, such as tests of normality and adjustment for multiple comparisons
☐	☒	A full description of the statistics including central tendency (e.g. means) or other basic estimates (e.g. regression coefficient) AND variation (e.g. standard deviation) or associated estimates of uncertainty (e.g. confidence intervals)
☐	☒	For null hypothesis testing, the test statistic (e.g. *F*, *t*, *r*) with confidence intervals, effect sizes, degrees of freedom and *P* value noted<br>*Give P values as exact values whenever suitable.*
☒	☐	For Bayesian analysis, information on the choice of priors and Markov chain Monte Carlo settings
☒	☐	For hierarchical and complex designs, identification of the appropriate level for tests and full reporting of outcomes
☒	☐	Estimates of effect sizes (e.g. Cohen's *d*, Pearson's *r*), indicating how they were calculated
☐	☒	Clearly defined error bars<br>*State explicitly what error bars represent (e.g. SD, SE, CI)*

Our web collection on statistics for biologists may be useful.

Software and code

Policy information about availability of computer code

Data collection	qPCR data were collected using a LightCycler96 (Roche). Flow cytometry data were acquired on a BD LSR II (BD Bioscience) with BD FACSDiva software.
Data analysis	GraphPad Prism 7 (GraphPad Software, Inc.) was used to prepare graphs and to perform statistical analyses. Flow cytometry data were analyzed using FlowJo v10 (Treestar, Inc.). Combination indices (CI) were calculated using CompuSyn (ComboSyn, Inc.) and indicated in median effect plots as a function of the bacteria fractions affected by the combinatorial peptide treatment.

For manuscripts utilizing custom algorithms or software that are central to the research but not yet described in published literature, software must be made available to editors/reviewers upon request. We strongly encourage code deposition in a community repository (e.g. GitHub). See the Nature Research guidelines for submitting code & software for further information.

Data

Policy information about availability of data

All manuscripts must include a data availability statement. This statement should provide the following information, where applicable:

- Accession codes, unique identifiers, or web links for publicly available datasets
- A list of figures that have associated raw data
- A description of any restrictions on data availability

The data that support the findings of this study are available from the corresponding author upon request.

Field-specific reporting

Please select the best fit for your research. If you are not sure, read the appropriate sections before making your selection.

☒ Life sciences ☐ Behavioural & social sciences ☐ Ecological, evolutionary & environmental sciences

For a reference copy of the document with all sections, see nature.com/authors/policies/ReportingSummary-flat.pdf

Life sciences study design

All studies must disclose on these points even when the disclosure is negative.

Sample size	No statistical methods were used to predetermine sample size, but our sample sizes are similar to those reported previously by us and by others
Data exclusions	No samples or animals were excluded from the analysis.
Replication	Experiments were replicated several times with reproducible results as indicated in each figure legend.
Randomization	Animals were randomly assigned to experimental groups at the beginning of experiments.
Blinding	Mouse experiments were performed by one person only. Therefore, blinding was not possible. In order to reduce bias treatment of mice was performed randomly and not in group order.

Reporting for specific materials, systems and methods

Materials & experimental systems

n/a	Involved in the study
☐	☒ Unique biological materials
☐	☒ Antibodies
☐	☒ Eukaryotic cell lines
☒	☐ Palaeontology
☐	☒ Animals and other organisms
☐	☒ Human research participants

Methods

n/a	Involved in the study
☐	☐ ChIP-seq
☐	☒ Flow cytometry
☐	☐ MRI-based neuroimaging

Unique biological materials

Policy information about availability of materials

Obtaining unique materials	All unique materials are available from the authors or can be produced by the methods described in the manuscript. Distribution of unique materials will be under a Material Transfer Agreement (MTA) where applicable.

Antibodies

Antibodies used	Mouse-monoclonal-APC-Cy7-CD45.2 clone 104, Biolegend, Cat#109824; Rat-monoclonal-PE-F4/80 clone BM8, BioLegend, Cat#123110; Rat-monoclonal-PerCP/Cy5.5-CD11b clone M1/70, BioLegend, Cat#101227; Armenian hamster-monoclonal-BV510TM-CD11c clone N418, BioLegend, Cat#117337; Rat-monoclonal-APC-Ly6G clone 1A8, BioLegend, Cat#127614; Rat-monoclonal-Pacific BlueTM-Ly6C clone HK1.4, BioLegend, Cat#128014; Rat-monoclonal-PE-CD19 clone 6D5, BioLegend,

	Cat#115508; Rat-monoclonal-Pacific-BlueTM-CD3 clone 17A2, BioLegend, Cat#100214; Mouse-monoclonal-PE/Cy7-NK1.1 clone PK136, BioLegend, Cat#108714; Rabbit-monoclonal-CAMP/LL37/FALL39/Cathelicidin, Novus Biologicals, Cat#NB100-98689; Rabbit-monoclonal-CXCL2 clone 16H3L10; Thermo Fisher Scientific, Cat# 701126; polyclonal-goat-Myeloperoxidase, R and D Systems, Cat#AF3667; mouse-monoclonal-LL-37/CAP-18 clone 1-1C12, HycultBiotech, cat# HM2071; anti-mosue IgG-HRP, Cell Signaling, cat# 7076S
Validation	All antibodies are from commercial sources and where previously validated by the vendors and can be found in the respective datasheet.

Eukaryotic cell lines

Policy information about cell lines

Cell line source(s)	HEK293T and HEK293T-TLR2 cells were obtained from Dorothee Kretschmer from the Interfaculty Institute for Microbiology and Infection Medicine Tübingen.
Authentication	None of the cell lines were authenticated.
Mycoplasma contamination	Routine laboratory mycoplasma detection showed no mycoplasma contamination.
Commonly misidentified lines (See ICLAC register)	None

Animals and other organisms

Policy information about studies involving animals; ARRIVE guidelines recommended for reporting animal research

Laboratory animals	C57BL/6 WT mice, female, 6-8 weeks old, purchased from Charles River Laboratories, Cat# 027C57BL/6; B6.129P2(SJL)-Myd88tm1.1Defr/J, female, 6-8 weeks old, University of Tübingen, Transgenic Facility; B6.129-Tlr2tm1(neo)Kir.129-TLR3tm1(neo)Aki.129P2-Tlr4tm1(neo)Aki.129-Tlr7tm1(neo)Aki.14.1-Tlr9tm1(neo)Aki, female, 6-8 weeks old, University of Tübingen, Transgenic Facility.
Wild animals	This study did not involve any wild animals.
Field-collected samples	This study did not involve any field-collected samples

Human research participants

Policy information about studies involving human research participants

Population characteristics	Healthy blood donation volunteers. Human foreskin donation volunteers.
Recruitment	Blood donation volunteers. Foreskin donation volunteers after routine circumcision.

ChIP-seq

Data deposition

☐ Confirm that both raw and final processed data have been deposited in a public database such as GEO.

☐ Confirm that you have deposited or provided access to graph files (e.g. BED files) for the called peaks.

Data access links *May remain private before publication.*	*For "Initial submission" or "Revised version" documents, provide reviewer access links. For your "Final submission" document, provide a link to the deposited data.*
Files in database submission	*Provide a list of all files available in the database submission.*
Genome browser session (e.g. UCSC)	*Provide a link to an anonymized genome browser session for "Initial submission" and "Revised version" documents only, to enable peer review. Write "no longer applicable" for "Final submission" documents.*

Methodology

Replicates	*Describe the experimental replicates, specifying number, type and replicate agreement.*
Sequencing depth	*Describe the sequencing depth for each experiment, providing the total number of reads, uniquely mapped reads, length of reads and whether they were paired- or single-end.*
Antibodies	*Describe the antibodies used for the ChIP-seq experiments; as applicable, provide supplier name, catalog number, clone name, and lot number.*
Peak calling parameters	*Specify the command line program and parameters used for read mapping and peak calling, including the ChIP, control and*

Peak calling parameters

index files used.

Data quality

Describe the methods used to ensure data quality in full detail, including how many peaks are at FDR 5% and above 5-fold enrichment.

Software

Describe the software used to collect and analyze the ChIP-seq data. For custom code that has been deposited into a community repository, provide accession details.

Flow Cytometry

Plots

Confirm that:

☒ The axis labels state the marker and fluorochrome used (e.g. CD4-FITC).

☒ The axis scales are clearly visible. Include numbers along axes only for bottom left plot of group (a 'group' is an analysis of identical markers).

☒ All plots are contour plots with outliers or pseudocolor plots.

☒ A numerical value for number of cells or percentage (with statistics) is provided.

Methodology

Sample preparation

To prepare single-cell suspensions, relevant dorsal skin area was transferred to PBS + 2% FBS (Biochrom/ Merck Millipore). Subcutaneous fat was removed using a razor blade and skin tissue was transferred into a 2 mL reaction tube containing digestion solution. Digestion solution contained 0.05 mg/ml DNase I (Roche) and 0.25 mg/ml Liberase (Roche) in RPMI-1640 Medium (Gibco/ life technologies). After scissor-mediated tissue disintegration digestion was performed for 1 h at 37 °C and stopped by addition of 100 µL of FCS (Biochrom/ Merck Millipore). Single cells were separated by using an 80 µm cell strainer (Greiner Bio-One). After washing in PBS + 2% FBS single cell suspensions were treated with TruStain fcXTM anti CD16/32 (BioLegend) and subsequently surface-stained.

Instrument

BD LSRII flow cytometer (BD Bioscience)

Software

BD FACSDiva software (BD Bioscience) was used for data acquisition. FlowJo software (Tree Star, Inc.) was used to analyze the data.

Cell population abundance

Describe the abundance of the relevant cell populations within post-sort fractions, providing details on the purity of the samples and how it was determined.

Gating strategy

Immune cells were gated by FSC-A/SSC-A and then doublet discrimination was performed first by FSC-H/FSC-A followed by SSC-H/SSC-A. Next, live CD45-positive cells were gated and then gating focused on quantifying cell specific populations: live CD45+ NK1.1+ NK-cells; live CD45+ CD19+ B cells; live CD45+ CD3+ T cells, live CD45+ CD11c+ dendritic cells; live CD45+ CD11b+ Ly6G+ Ly6C+ Neutrophils; live CD45+ CD11b+ Ly6C+ Monocytes; live CD45+ CD11b+ F4/80+ Macrophages;

☒ Tick this box to confirm that a figure exemplifying the gating strategy is provided in the Supplementary Information.

Magnetic resonance imaging

Experimental design

Design type

Indicate task or resting state; event-related or block design.

Design specifications

Specify the number of blocks, trials or experimental units per session and/or subject, and specify the length of each trial or block (if trials are blocked) and interval between trials.

Behavioral performance measures

State number and/or type of variables recorded (e.g. correct button press, response time) and what statistics were used to establish that the subjects were performing the task as expected (e.g. mean, range, and/or standard deviation across subjects).

Acquisition

Imaging type(s)

Specify: functional, structural, diffusion, perfusion.

Field strength

Specify in Tesla

Sequence & imaging parameters

Specify the pulse sequence type (gradient echo, spin echo, etc.), imaging type (EPI, spiral, etc.), field of view, matrix size, slice thickness, orientation and TE/TR/flip angle.

Area of acquisition

State whether a whole brain scan was used OR define the area of acquisition, describing how the region was determined.

Diffusion MRI ☐ Used ☐ Not used

Preprocessing

Preprocessing software	*Provide detail on software version and revision number and on specific parameters (model/functions, brain extraction, segmentation, smoothing kernel size, etc.).*
Normalization	*If data were normalized/standardized, describe the approach(es): specify linear or non-linear and define image types used for transformation OR indicate that data were not normalized and explain rationale for lack of normalization.*
Normalization template	*Describe the template used for normalization/transformation, specifying subject space or group standardized space (e.g. original Talairach, MNI305, ICBM152) OR indicate that the data were not normalized.*
Noise and artifact removal	*Describe your procedure(s) for artifact and structured noise removal, specifying motion parameters, tissue signals and physiological signals (heart rate, respiration).*
Volume censoring	*Define your software and/or method and criteria for volume censoring, and state the extent of such censoring.*

Statistical modeling & inference

Model type and settings	*Specify type (mass univariate, multivariate, RSA, predictive, etc.) and describe essential details of the model at the first and second levels (e.g. fixed, random or mixed effects; drift or auto-correlation).*
Effect(s) tested	*Define precise effect in terms of the task or stimulus conditions instead of psychological concepts and indicate whether ANOVA or factorial designs were used.*

Specify type of analysis: ☐ Whole brain ☐ ROI-based ☐ Both

Statistic type for inference (See Eklund et al. 2016)	*Specify voxel-wise or cluster-wise and report all relevant parameters for cluster-wise methods.*
Correction	*Describe the type of correction and how it is obtained for multiple comparisons (e.g. FWE, FDR, permutation or Monte Carlo).*

Models & analysis

n/a	Involved in the study
☐	☐ Functional and/or effective connectivity
☐	☐ Graph analysis
☐	☐ Multivariate modeling or predictive analysis

Functional and/or effective connectivity	*Report the measures of dependence used and the model details (e.g. Pearson correlation, partial correlation, mutual information).*
Graph analysis	*Report the dependent variable and connectivity measure, specifying weighted graph or binarized graph, subject- or group-level, and the global and/or node summaries used (e.g. clustering coefficient, efficiency, etc.).*
Multivariate modeling and predictive analysis	*Specify independent variables, features extraction and dimension reduction, model, training and evaluation metrics.*
